# The microbiota characterizing huge carbonatic moonmilk structures and its correlation with preserved organic matter

**DOI:** 10.1186/s40793-024-00562-9

**Published:** 2024-04-24

**Authors:** Daniele Ghezzi, Nicasio Tomás Jiménez-Morillo, Lisa Foschi, Eva Donini, Veronica Chiarini, Jo De Waele, Ana Zélia Miller, Martina Cappelletti

**Affiliations:** 1 Department of Pharmacy and Biotechnology, University of Bologna, Via Irnerio 42, Bologna, 40126 Italy; 2 MED—Mediterranean Institute for Agriculture, Environment and Development, University of Évora, Pólo da Mitra Apartado 94, Évora, 7006-554 Portugal; 3 Instituto de Recursos Naturales y Agrobiologia de Sevilla (IRNAS-CSIC), Av. de la Reina Mercedes, 10, Sevilla, 41012 Spain; 4 Department of Geosciences, University of Padova, via Gradenigo 6, Padua, 35131 Italy; 5 Department of Biological, Geological, and Environmental Sciences, University of Bologna, Via Zamboni 67, Bologna, 40126 Italy; 6 HERCULES Laboratory, University of Évora, Largo dos Colegiais 2, Évora, 7004-516 Portugal

**Keywords:** Cave microbiota, Moonmilk, Speleothem, Organic matter, Carbonatic rock, *Methylomirabilota*

## Abstract

**Background:**

Moonmilk represents complex secondary structures and model systems to investigate the interaction between microorganisms and carbonatic rocks. Grotta Nera is characterized by numerous moonmilk speleothems of exceptional size hanging from the ceiling, reaching over two meters in length. In this work we combined microbiological analyses with analytical pyrolysis and carbon stable isotope data to determine the molecular composition of these complex moonmilk structures as well as the composition of the associated microbiota.

**Results:**

Three moonmilk structures were dissected into the apical, lateral, and core parts, which shared similar values of microbial abundance, richness, and carbon isotopes but different water content, microbiota composition, and organic matter. Moonmilk parts/niches showed higher values of microbial biomass and biodiversity compared to the bedrock (not showing moonmilk development signs) and the waters (collected below dripping moonmilk), indicating the presence of more complex microbial communities linked to carbonate rock interactions and biomineralization processes. Although each moonmilk niche was characterized by a specific microbiota as well as a distinct organic carbon profile, statistical analyses clustered the samples in two main groups, one including the moonmilk lateral part and the bedrock and the other including the core and apical parts of the speleothem. The organic matter profile of both these groups showed two well-differentiated organic carbon groups, one from cave microbial activity and the other from the leaching of vascular plant litter above the cave. Correlation between organic matter composition and microbial taxa in the different moonmilk niches were found, linking the presence of condensed organic compounds in the apical part with the orders *Nitrospirales* and *Nitrosopumilales*, while different taxa were correlated with aromatic, lignin, and polysaccharides in the moonmilk core. These findings are in line with the metabolic potential of these microbial taxa suggesting how the molecular composition of the preserved organic matter drives the microbiota colonizing the different moonmilk niches. Furthermore, distinct bacterial and archaeal taxa known to be involved in the metabolism of inorganic nitrogen and C_1_ gases (CO_2_ and CH_4_) (*Nitrospira*, *Nitrosopumilaceae*, *Nitrosomonadaceae*, *Nitrosococcaceae*, and novel taxa of *Methylomirabilota* and *Methanomassiliicoccales*) were enriched in the core and apical parts of the moonmilk, probably in association with their contribution to biogeochemical cycles in Grotta Nera ecosystem and moonmilk development.

**Conclusions:**

The moonmilk deposits can be divided into diverse niches following oxygen and water gradients, which are characterized by specific microbial taxa and organic matter composition originating from microbial activities or deriving from soil and vegetation above the cave. The metabolic capacities allowing the biodegradation of complex polymers from the vegetation above the cave and the use of inorganic nitrogen and atmospheric gases might have fueled the development of complex microbial communities that, by interacting with the carbonatic rock, led to the formation of these massive moonmilk speleothems in Grotta Nera.

**Electronic Supplementary Material:**

The online version contains supplementary material available at 10.1186/s40793-024-00562-9.

## Background

Caves are natural underground openings with lengths ranging from a few to thousands of meters characterized by the absence of sunlight, a limited supply of nutrients, a rather stable temperature, high humidity levels, and higher partial pressure of CO_2_ compared to the external environment [1, 2]. In caves, microorganisms interact with the inorganic minerals contained in the rocks typically mediating their solubilization and precipitation leading to speleothem formation of different kinds and on different rock substrates [3–5]. Microorganisms typically organize themselves in complex biological formations, which are visible as biofilms or biodeposits [6–8]. Cave microbes of both bacterial and archaeal domains support the ecosystem dominating primary production and feeding biogeochemical cycles through chemolithotrophic activities such as the anaerobic iron and ammonium oxidation, methanogenesis, sulfate and iron reduction, and hydrogenotrophic denitrification, and the aerobic oxidation of methane, ammonia, carbon monoxide, nitrite, iron, hydrogen, and sulfur [9–11].

Carbonate deposition and dissolution are common processes in caves, and the participation of microorganisms in these processes has been reported [8, 12–14]. Limestone caves often contain various types of speleothems, such as stalactites and stalagmites, which are mainly composed of calcium carbonate (CaCO_3_) [15]. Moonmilk is a white deposit observed on walls and ceilings of limestone caves worldwide, with various textures depending on the water content, but generally soft like toothpaste when wet, and powdery when dry [16, 17]. Moonmilk is generally composed of various calcite crystal morphologies, most often with micrometer- or nanometer length crystals or calcite fibers similar to microbial filaments [14–16]. An exception is represented by moonmilk deposits exclusively made of gypsum that have been described in sulfidic caves [8]. The full mechanisms of calcite moonmilk formation remains poorly understood; however, several morphological and functional observations have associated this speleothem formation with biological processes leading to (i) direct precipitation of calcite carried out by microbial metabolism and enzymes and/or (ii) indirect precipitation of the mineral due to the function of microbial surfaces as nucleation sites [14, 16, 18, 19]. In line with this, several studies showed that, within the bacterial communities involved, there is a great variety of bacteria belonging to *Pseudomonadota* and *Actinomycetota* capable of influencing the deposition of calcium carbonate, regardless of the type of deposit. Other phyla frequently found in calcite moonmilks are *Acidobacteriota* and *Chloroflexota* [20, 21] whose role in this speleothem formation is still elusive due to the “unculturability” of members of this phylum and the lack of specific metagenomic analyses.

**Fig. 1:**
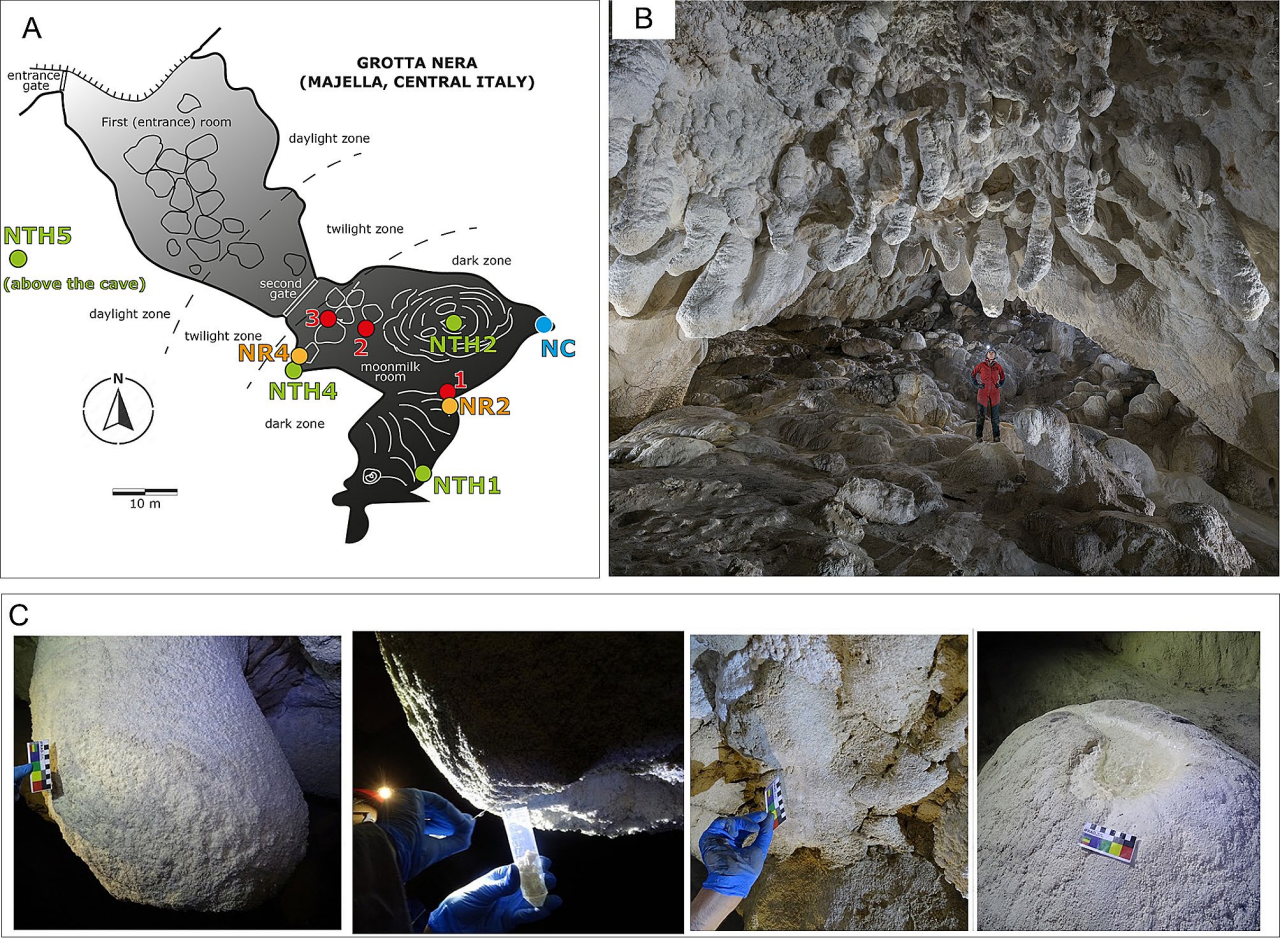
**(A)** Cave map showing the sampling sites for microbiology and biogeochemistry analyses (1, 2, 3) as well as the sites in which the detectors were located for CO_2_ (NC dots), temperature (NTH dots), and radon (NR dots) monitoring. **(B)** Picture inside Grotta Nera showing the outstanding moonmilk structures characterizing the cave (Picture courtesy of UPIX – Fotografia ipogea). **(C)** Representative pictures of the different samples that were collected and studied. From the left: the moonmilk structure, the apical part of the moonmilk, the bedrock, and the water body

Grotta Nera (Black Cave), although of rather modest dimensions, is one of the most important caves in the Majella National Park (Abruzzo, Italy) presenting outstanding ivory-white moonmilk speleothems in the form of stalactites, stalagmites, and flowstones [22] (Fig. 1). These calcite moonmilk structures are unique in the world in terms of both abundance and dimension [23, 24]. These structures were first described by Forti and Rossi (2003) as flat-bottomed speleothems whose development seems to be related to strong evaporation-condensation processes caused by complex air flows within the cave room. Cacchio et al. (2014) provided first indications on the presence of microorganisms in the moonmilk structures that were isolated in the laboratory and some of them showed the ability to solubilize calcium carbonate.

In this study, we provide an in-depth characterization of the moonmilk deposits in Grotta Nera in correlation with the environmental parameters present inside the cave together with the results of carbon stable isotope, 16 S rRNA metabarcoding, and analytical pyrolysis (pyrolysis gas chromatography/mass spectrometry) to get insights into the cave environmental conditions, the microbiota, and the organic molecules characterizing the different niches composing these complex structures derived from the microbial interaction with carbonatic rock substrates.

## Methods

### Cave description

Grotta Nera is located at around 1400 m a.m.s.l. in the territory of Pennapiedimonte, on the eastern slope of the Majella Mountains, in the Feudo Ugni Natural Reserve (Majella National Park, Abruzzo, Italy). Although it is a relatively small cave (only 110 m long), it is well-known for its spectacular speleothems. The area of the cave is located in a mixed-deciduous forest characterized by large seasonal temperature shifts (mean annual between 8 and 17 °C, with mean minimal and maximal values ranging from − 2 to + 27 °C [25] and high mean precipitation of around 1400 mm (part of which is snow). The Majella Massif hosts several karst caves [26], some of which are popular tourist attractions (i.e., Grotta del Cavallone [27–29]). Access to the Grotta Nera is strictly regulated, to preserve the cave and its peculiar speleothems, avoiding possible perturbations due to speleotourism. Starting from the wide entrance, an ascending rocky track inside the cave leads through the first large room, mostly illuminated from the outside. At the end of this large space, a smaller slightly ascending passage, equipped with a gate, takes to the main room of the cave. This narrow passage, which forms a threshold for the incoming and outflowing air, prevents the external light to penetrate further into the cave. This second room, which develops also to the south along a short ascending branch and some narrow passages in the middle of speleothems, is entirely dark, except for the areas close to the gate, and hosts the moonmilk speleothems. From a speleogenetic point of view, both entrance and inner chambers are characterized by significant enlargement through gravitational collapse of blocks from the roof, and their floor is largely covered with large limestone slabs. Neither water flows nor water-transported deposits have been reported; only a moderate dripping in the inner zone of the cave has been observed [23].

### Temperature, carbon dioxide and radon analyses

All sensors, but the one measuring the outside conditions which was located above the cave in the shadow of a rock outcrop (NTH5 in **Additional file 1: Fig. S1**), were placed in the second room of the cave, where light conditions are dark and all moonmilk samples were collected. The air temperature in the entrance room is strongly affected by the climatic conditions outside, thus by strong seasonal variability [23]. The temperatures in the innermost rooms (stations NTH in Fig. 1) were monitored from June 2019 to June 2020 with Tinytag TGP 4505 loggers (Gemini, Chichester, UK), with an accuracy of 0.3 °C. CO_2_ concentrations in the air were recorded (NC in Fig. 1) with CM-0018-AA CO2meters (Ormond Beach, Florida) measuring up to 1% CO_2_ (10,000 ppm) with an accuracy of 30 ppm including an RH sensor (0-100% RH) with 3% accuracy. Radon concentration was checked on a three-monthly basis using passive CR-39 solid state nuclear track detectors placed in several points in the cave (NR2 and NR4 in Fig. 1) (sensitivity: 2.69 kBq h m^− 3^; minimum detectable activity for 1-year long measurement: 3 Bq m^− 3^). The analyses have been carried out by an automatic track reading developed by U-Series Srl (Bologna, Italy). The accuracy of the measurements is periodically checked and are normally better than 10%.

### Moonmilk sampling

In this work, we collected three types of samples from three different moonmilk speleothems located in different dark cave zones (i.e., the most inner zone, the middle zone, and the zone proximal to the entrance; points indicated as 1, 2, and 3 in Fig. 1, respectively). The three types of samples in each moonmilk speleothem were representative of the apical, the lateral, and the core part of the speleothem under analysis (the latter collected by coring 7–9 cm inside the speleothem) (Fig. 1). Additionally, we collected samples from three bedrock areas proximal to each moonmilk location (i.e., the cave area close to the moonmilk but not presenting sign of secondary mineral deposit or speleothem development) (Fig. 1**)** and three water pools immediately below each speleothem constituted by the water dripping from it. The water content of all samples was determined by measuring the water loss after drying them at 80 °C to a constant weight. All the sample descriptions and the sample codes are indicated in Table 1.

**Table 1:** Description of the samples collected in Grotta Nera, collection sites, sequencing results, pH values, and water content

Sampling site	Sample ID	Description	# filtered reads	# ASV^a^	pH	% water
Innermost zone of the cave	AM-1	Apical part of the moonmilk	19,423	673	6.5	76.4
	LM-1	Lateral part of the moonmilk	24,382	698	6	40.3
	CM-1	Core part of the moonmilk	17,363	504	6.5	55.1
	Rk-1	Bedrock	18,095	456	5.5	27.8
	Wt-1	Water on the floor^b^	19,269	284	6.5	99.5
Middle zone of the cave	AM-2	Apical part of the moonmilk	17,059	528	6.5	80.2
	LM-2	Lateral part of the moonmilk	20,381	458	6	37.6
	CM-2	Core part of the moonmilk	17,108	523	6.5	53.2
	Rk-2	Bedrock	7,598	222	5.5	25.2
	Wt-2	Water on the floor^b^	24,904	89	6.5	99.1
Proximal to cave entrance (in the dark)	AM-3	Apical part of the moonmilk	18,706	571	6.5	84.6
	LM-3	Lateral part of the moonmilk	18,484	456	6	45.0
	CM-3	Core part of the moonmilk	14,204	407	6.5	69.0
	Rk-3	Bedrock	16,145	474	5.5	43.1
	Wt-3	Water on the floor^b^	6,444	19	6.5	99.8
Soil above the cave	Soil	Forest soil	4,654	135	5.5	34.5

^a^ Number of Amplicon Sequence Variants (ASV) obtained through DADA2 software

^b^ Water samples were collected from water bodies storing the dripping water from the moonmilk analyzed in each of the three cave zones

### Carbon stable isotope analysis

The carbon isotope composition ratios (^13^C/^12^C) of the different moonmilk niches/parts, bedrock, and aboveground soil samples were determined using an elemental analyzer Flash 2000 HT (Thermo Scientific, Bremen, Germany) coupled by a ConFlo IV interface unit to a continuous flow Delta V Advantage isotope ratio mass spectrometer (IRMS) (Thermo Scientific, Bremen, Germany), as described in [30]. Carbon isotope ratios “𝛿^13^C” are reported in the standard notation (‰) from appropriate standards recognized by the International Atomic Energy Agency (IAEA). Selected standard reference materials were used for this analysis: (i) cellulose (IAEA-CH-3, 𝛿^13^C = -24.7‰); (ii) sucrose (IAEA-CH-6, 𝛿^13^C = -10.5‰), and (iii) caffeine (IAEA-600, 𝛿^13^C = -27.8‰). The standard deviation of bulk 𝛿^13^C was ± 0.1‰. Each sample was measured in duplicate (*n* = 2).

### Analytical pyrolysis

Pyrolysis gas chromatography/mass spectrometry (Py-GC/MS) is a fast and reproducible technique widely used for the molecular characterization of organic matter [30, 31]. The technique involves a thermolytic degradation of macromolecules into small fragments that can be separated and identified. Py-GC/MS was performed using a double-shot pyrolyzer (Frontier Laboratories, model 2020i), working at 500 °C, attached to a Shimadzu GC2010 gas chromatographer with a Shimadzu GCMS-QP2010 Plus mass spectrometer (Shimadzu) working in negative mode at 70 eV. A capillary column Zebron-ZB-5HT (30 m length, 0.25 mm internal diameter, 0.10 μm film thickness) was used for separation, with helium as carrier gas, adjusted to a flow rate of 1.2 mL min^− 1^. The splitless injector operated at a temperature of 250 °C. The chromatographic ramp of temperature was previously described by [32]: 50 °C for 1 min followed by an increase to 100 °C at 30 °C min^− 1^, from 100 to 300 °C at 10 °C min^− 1^, and stabilized at 300 °C for 10 min. The mass spectrometer was programmed to acquire data between 40 and 850 m/z. Compound assignment was achieved by monitoring diagnostic ions for the main homologous series (m/z 57 for *n*-alkanes), via low-resolution MS and via comparison with published and stored (NIST and Wiley libraries) data. Diketopiperazine compounds were integrated monitoring the specific combination of ion fragmentations [33]. The average chain length index of alkane compounds from different samples was calculated following the equation proposed by Poynter and Eglinton (1990) [34]. Surface density van Krevelen plots were constructed as described by Jiménez-Morillo et al. [35] to identify the most abundant organic compounds present in each sample. Briefly, the contour diagrams were obtained by subtracting the compound abundances from pairs of samples corresponding to the different parts of moonmilk speleothems and bedrocks and represented in the x,y plane defined by the atomic H/C (y) and O/C (x) ratio of each molecular formula.

### DNA extraction and Illumina sequencing

Three DNA extractions were conducted for each type of cave sample (moonmilk apical, lateral, and core parts, bedrock, and water) and for the soil collected above Grotta Nera using the DNeasy PowerSoil Kit (Qiagen) according to the manufacturer’s protocol with slight modifications as previously described [36]. The extracted DNAs from the replicates of each type of sample were pooled to be used as template for PCR amplification targeting the V4 and V5 hypervariable regions of the 16S rRNA gene using the primer pair 515F (5’-GTGYCAGCMGCCGCGGTA-3’) and 907R (5’-CCCCGYCAATTCMTTTRAGT-3’) [37] modified with an Illumina adaptor sequence at the 5’ end. PCR reactions were performed in a final volume of 50 µL containing 10 ng of total DNA, primers 500 nM, 1x Takara Ex Taq buffer with MgCl_2_, dNTPs mix 200 µM, Takara Ex Taq Polymerase 0.5 U. The thermocycling program included 1 cycle at 95 °C for 10 s, 30 cycles at 95 °C for 10 s, 56 °C for 30 s, 72 °C for 30 s, and a final extension at 72 °C for 2 min. Amplicons were submitted to the library preparation and Illumina MiSeq sequencing platform for indexing and pair-end sequencing (2 × 250 bp; reagent kit, v2) at the sequencing service BMR Genomics SRL. The demultiplexed and primer-clipped reads were trimmed based on quality and length, and further dereplicated, denoised, merged, and checked for chimeras by using Qiime2 version 2018.4 [38]. Reads were processed into Amplicon Sequence Variants (ASVs) using the DADA2 package version 1.14 as described in [39]. The taxonomic assignment of the resulting ASVs was performed by querying the ASVs against SILVA SSU database version r138.1 [40]. We manually modified the nomenclature of the prokaryotic phyla according to the latest updates from the International Committee on Systematics of Prokaryotes [41]. Eukaryotic, mitochondrial, and chloroplast sequences were removed from further analyses. The Illumina sequencing raw data were deposited in the Sequence Read Archive of NCBI under accession number PRJNA1052665.

### Quantitative PCR

Quantitative PCR was conducted on the CFX96 Real-Time PCR Detection System (Bio-Rad Laboratories Inc., Hercules, USA) by using SYBR green-based reactions. The quantification of the bacterial and archaeal 16 S rRNA genes was performed in triplicate. The primer sets 926 F-1062R [42] and 519 F-806R [43] were used to amplify the bacterial and the archaeal 16 S rRNA genes, respectively, in a 10 µL qPCR reaction mix containing: 1 µL of the total extracted DNA, primers 300 nM each, 1x AceQ qPCR SYBR Green Master Mix (Vazyme Biotech Co., Nanjing, China), using the thermocycling conditions: 95 °C for 5 min, 40 cycles of 95 °C for 10 s and 60 °C for 30 s. Serial dilutions (across six orders of magnitude, i.e., 10^1^-10^6^) of 16 S rRNA gene PCR products from *Escherichia coli* and *Nitrososphaera viennensis* were used as standards for the bacterial and the archaeal 16 S rRNA genes, as previously described [43]. The standard curves for *E. coli* and *N. viennensis* showed correlation coefficients (R^2^) > 0.99.

### Statistical and phylogenetic analyses

One-way analysis of variance (ANOVA) was carried out comparing means using the Tukey test, *P* = 0.05 using the Statgraphics Centurion XVIII software, to evaluate significant differences among 𝛿^13^C values for different moonmilk speleothems, bedrock, and aboveground soil samples.

Alpha diversity estimates of the microbial communities were generated by using the Shannon, Simpson’s, Chao1, and Evenness indexes with statistical significance determined by ANOVA in Calypso [44]. Beta-diversity was assessed using Principal Coordinates Analysis (PCoA) method with Bray-Curtis distance and PERMANOVA statistical method in Microbiome Analyst [45]. MicrobiomeAnalyst was also used to define the “core microbiome” that corresponds to the abundant microbial taxa present in all the samples representative of each type of matrix (sample prevalence 75%, relative abundance 0.01%). Linear discriminant analysis effect size (LEfSe) was used to evaluate the differences between the prokaryotic communities present in the different groups of samples (representative of the different matrix/types of samples present in Grotta Nera and the different moonmilk niches). Significant features were considered for an adjusted *p*-value < 0.05. Microbial community profiles of the 16 S rRNA gene sequences and graphical visualization were conducted using Microbiome Analyst with parameters for counter filters (0), prevalence in samples (10%), and inter-quartile variance filter. Data were normalized using the Total Sum Scaling (TSS) method.

An unrooted phylogenetic tree was built with the neighbor-joining method, maximizing the likelihood with a gamma model distribution, and using the ASVs representative of each sample groups and the most closely related sequences retrieved from the NCBI database (Best Blast Hits). MEGA11 [46] was used to construct phylogenetic trees based on ClustalW sequences alignment and neighbor-joining clustering method with 1000 non-parametric bootstrap replicates (model: Jukes-Cantor; rates among site: uniform rates; gap/missing data treatment: pairwise deletion). iTOL was used to visualize the phylogenetic tree.

## Results

### Analysis of temperature and CO_2_ in the cave

Sensor locations are displayed in Fig. 1, and results of the monitoring are shown in **Additional file 1: Fig. S1**. The innermost temperature logger NTH1 recorded minimum values of 5.8 °C and maximum values of 15.8 °C, with an annual mean of 8.7 °C. The station NR4 located closer to the second entrance gate and placed close to the ground, in one of the lowest and coldest locations in the cave, registered values between 5.2 and 12.8 °C, recording a mean annual value of 7.8 °C. The sensor NTH2, placed close to the ceiling in the central part of the room reported higher values, from 6.1 to 14.5 °C with a mean annual of 9.9 °C. This second room is still greatly influenced by the outside meteoric conditions, with daily temperature oscillations of up to 6 to 8 °C, and mean annual variations between winter and summer of 4 to 5 °C. From late autumn to mid spring the cave temperature is higher than that registered outside, whereas during summer it is up to 5 °C colder. The temperature, however, never gets lower than 5 °C, so freezing conditions are never met. It is interesting to note that on average the cave temperature close to the roof is 2 to 3 °C warmer than at the floor. Based on these temperatures and the morphology of the cave, with an ascending profile and a threshold at the second gate leading into the descending inner parts of the cave, it can be stated that warmer and moister outside air entering the cave can cause condensation to occur along the roof only during the summer months (from the start of June to end of August).

The CO_2_ concentration in the cave air, measured at ground level (NC in Fig. 1) ranged between 600 and 3700 ppm, with a mean annual value of 1100 ppm. Winter values were generally at least half those measured in late spring and summer, mostly reflecting lower biological activity in the soil above the cave, and thus less CO_2_ conveyed through dripping waters into the cave. Radon concentration ranged between 80 and 1600 Bq m^− 3^ with higher values in the inner part of the cave (NR2), reflecting less air circulation here.

### Carbon isotope composition

**Fig. 2:**
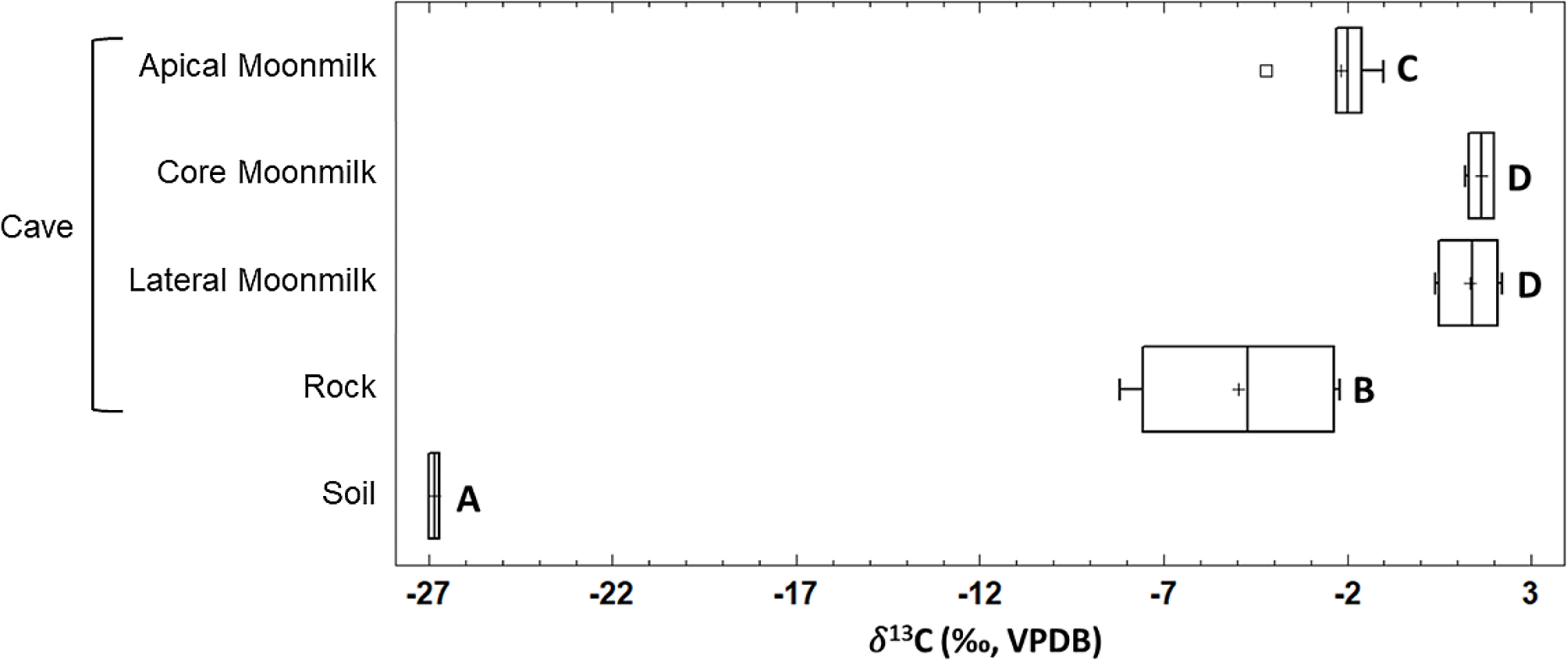
Boxplots of the bulk carbon (𝛿^13^C) isotope composition of moonmilk speleothem parts, bedrock, and aboveground soil from Grotta Nera. Boxplots show the ranges, lower and upper quartiles (Q1, Q3), and the median (Q2). Boxplots with different capital letters indicate statistical differences (ANOVA; Means compared using Tukey test, *p* = 0.05)

The carbon isotope composition of the moonmilk, bedrock and surface soil samples of Grotta Nera are depicted in **Additional file 1: Table S1**. The average 𝛿^13^C values of the soil sample (-26.9 ± 0.2‰) was typical of C_3_ vegetation plants and significantly lower than the cave (moonmilk and bedrock) samples. The comparison of the 𝛿^13^C values of moonmilk and bedrock samples among sampling locations (inner, middle, and outer zones) shows no remarkable differences (**Additional file 1: Table S1**). However, a noticeable difference of the mean 𝛿^13^C values between the moonmilk parts (apical, core, and lateral) and the bedrock samples is observed. The one-way ANOVA of the 𝛿^13^C values depicted in Fig. 2 clearly demonstrates these significant differences (*p* < 0.05), being the bedrock the most ^13^C-depleted sample (-4.9‰). In contrast, the lateral and core parts of the moonmilk samples showed no significant differences in carbon isotope composition (1.3‰ and 1.6‰, respectively).

### Molecular composition of the organic matter in moonmilk speleothems, cave bedrock, and soil above the cave

**Fig. 3:**
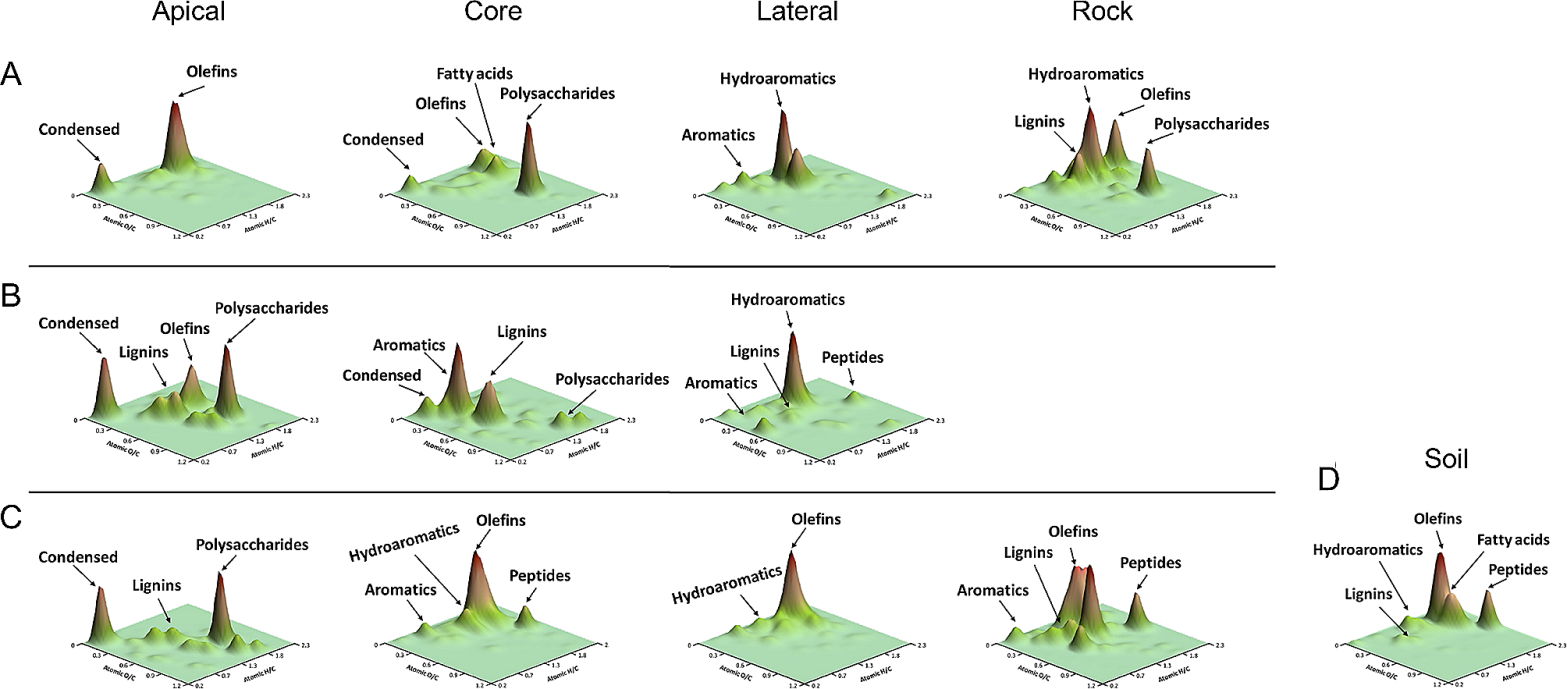
3D van Krevelen diagrams illustrating the molecular composition of the organic matter preserved in the different parts (apical, lateral, and core) of the moonmilk speleothem and bedrock located in: **(A)** the innermost zone of the cave; **(B)** the middle zone of the cave; and **(C)** the proximity of the cave entrance. The molecular composition of the soil sample (collected above the cave) is indicated in **(D)**. The organic compounds released by Py-GC/MS were represented in the space defined by their atomic H/C and O/C ratios

The molecular composition of the organic fraction preserved in the moonmilk samples, and the bedrock samples was compared using 3D van Krevelen (Fig. 3). As a result, visible differences in organic matter composition were observed between the parts/niches composing each moonmilk (apical, lateral, and core) and the corresponding bedrock samples. In regard with the moonmilk located inside the cave, the proximal bedrock showed the most complex organic composition, with predominance of lipid-like (olefins, unsaturated lipids, and fatty acids; 1.5 > H/C < 2.0 and 0.1 > O/C < 0.4), hydroaromatic (1.3 > H/C < 2.0 and O/C < 0.3), lignin-like (0.5 > H/C < 1.8 and 0.1 > O/C < 0.7), and polysaccharide (H/C > 1.5 and O/C > 0.8) compounds. In contrast, the apical moonmilk sample showed the lowest diversity of organic families, mainly represented by condensed-like (H/C < 0.5 and O/C < 0.1) and lipid-like compounds. In the lateral part of the moonmilk speleothems, the preserved organic matter was mainly composed of hydroaromatic (1.3 > H/C < 2.0 and O/C < 0.3) and aromatic-like (0.7 > H/C < 1.3 and O/C < 0.3) compounds. Finally, a big portion of the organic fraction preserved in the core of the speleothem was represented by polysaccharides (Fig. 3), while lipids (such as olefins and fatty acids) and condensed-like compounds were present but with a much lower abundance. In regard with the moonmilk speleothem in the middle zone of the cave, the apical part was mainly composed of polysaccharides, lipids, and lignin-like (1.0 > H/C < 1.5 and O/C > 0.8) compounds and it also showed a remarkable proportion of polycyclic aromatic hydrocarbons (H/C < 0.5 and O/C < 0.1). The lateral part was characterized by a high proportion of hydroaromatic molecules, followed by lignin, aromatic- and protein-like (such as, peptides, H/C > 2 and 0.5 > O/C < 0.7) compounds. The sample obtained from the core of the moonmilk showed an organic phase rich in molecules derived from methoxyphenols (lignin), aromatics, and polysaccharides. In regard with the moonmilk speleothem collected closer to the cave entrance, the bedrock sample showed a chemically complex organic phase, mainly composed of materials derived from lipid-, protein-, lignin-, and aromatic-like compounds. The organic matter preserved in the apical part was dominated by polysaccharides and condensed-like compounds, with a relative high contribution of lignin molecules. The lateral sample of the moonmilk showed a chemically poor organic fraction, mainly composed of olefin molecules (lipids), while the core part revealed a complex organic fraction dominated by different compound families, such as lipids, proteins, hydroaromatics, and aromatic-like compounds.

The molecular composition of the organic matter in the surface soil was also characterized to infer the potential source of the organic matter preserved in the speleothems (Fig. 3). The soil organic matter was mainly composed of lipid-like compounds (i.e., olefins and fatty acids), which are present in plant waxes. Soil also showed a remarkable abundance of protein-like, hydroaromatic, and lignin-like compounds.

The detailed study of the different families of organic compounds, such as hydrocarbons (fatty acids and *n*-alkanes) and peptides (diketopiperazines), was carried out (**Additional file 1: Table S2 and Table S3)**. It was observed that the lipid profile obtained in almost all samples showed a bimodal tendency (**Additional file 1: Table S2**), i.e., a maximum corresponding to the short-chain *n*-alkanes (C_13_, tridecane), corresponding to possible microbial activity, and another maximum corresponding to long-chain *n*-alkanes (C_25 − 26_), generally associated with higher plants [47, 48]. In addition, all samples showed the presence of the fatty acids *n*-hexadecanoic (C_16:0_) and octadecanoic (C_18:0_) acids, as well as the triterpene C_30_H_50_ (named squalene), which is a typical precursor of secondary metabolites produced by various living organisms. In the speleothem samples located in the innermost zone of the cave, the bedrock and the lateral part of the moonmilk samples showed the highest proportion of long-chain *n*-alkanes, while the core samples presented a remarkable depletion of these alkyl compounds. On the other hand, in the middle zone of the cave, the apical part of the moonmilk solely showed mid-chain *n*-alkanes (C_23_, tricosane), while the core moonmilk part was dominated by long-chain *n*-alkanes (> C_23_). The moonmilk and bedrock samples located closest to the cave entrance showed almost the same trend as the samples collected in the middle zone of the cave, with the core part showing a remarkable high proportion of long-chain *n*-alkanes (**Additional file 1: Table S2**). The lateral moonmilk part and bedrock samples located in the innermost zone of the cave and in the proximity of the cave entrance **(Additional file 1: Table S3)** also showed a remarkable abundance of diketopiperazine compounds, which are natural products of microbial origin.

### Microbial abundance and alpha-diversity

Quantitative PCR results indicated that the moonmilk samples have a higher abundance of the bacterial 16 S rRNA gene compared to the cave bedrock and waters (**Additional file 1: Fig. S2**). The lateral and apical parts of moonmilk showed the highest amount of bacterial biomass (around 10^8^ copies g^–1^), while it declined going to the moonmilk core (around 10^6^ copies g^–1^). In regard with archaeal sequences, the abundance of the archaeal 16 S rRNA gene in the moonmilk samples was lower than the bacterial abundance by 1–3 orders of magnitude. Conversely, no archaeal 16 S rRNA genes were detectable in the other cave samples, indicating the very low abundance or absence of archaeal members in the cave water and bedrock.

Illumina sequencing provided, after quality control, a total of 264,219 reads that were clustered in 4,683 ASVs (4,458 bacterial ASVs and 219 archaeal ASVs) through DADA2 (Table 1). The rarefaction curves showed that the sequencing depth was enough to fully describe the prokaryotic diversity present in the samples under analysis (**Additional file 1: Fig. S3**).

**Fig. 4:**
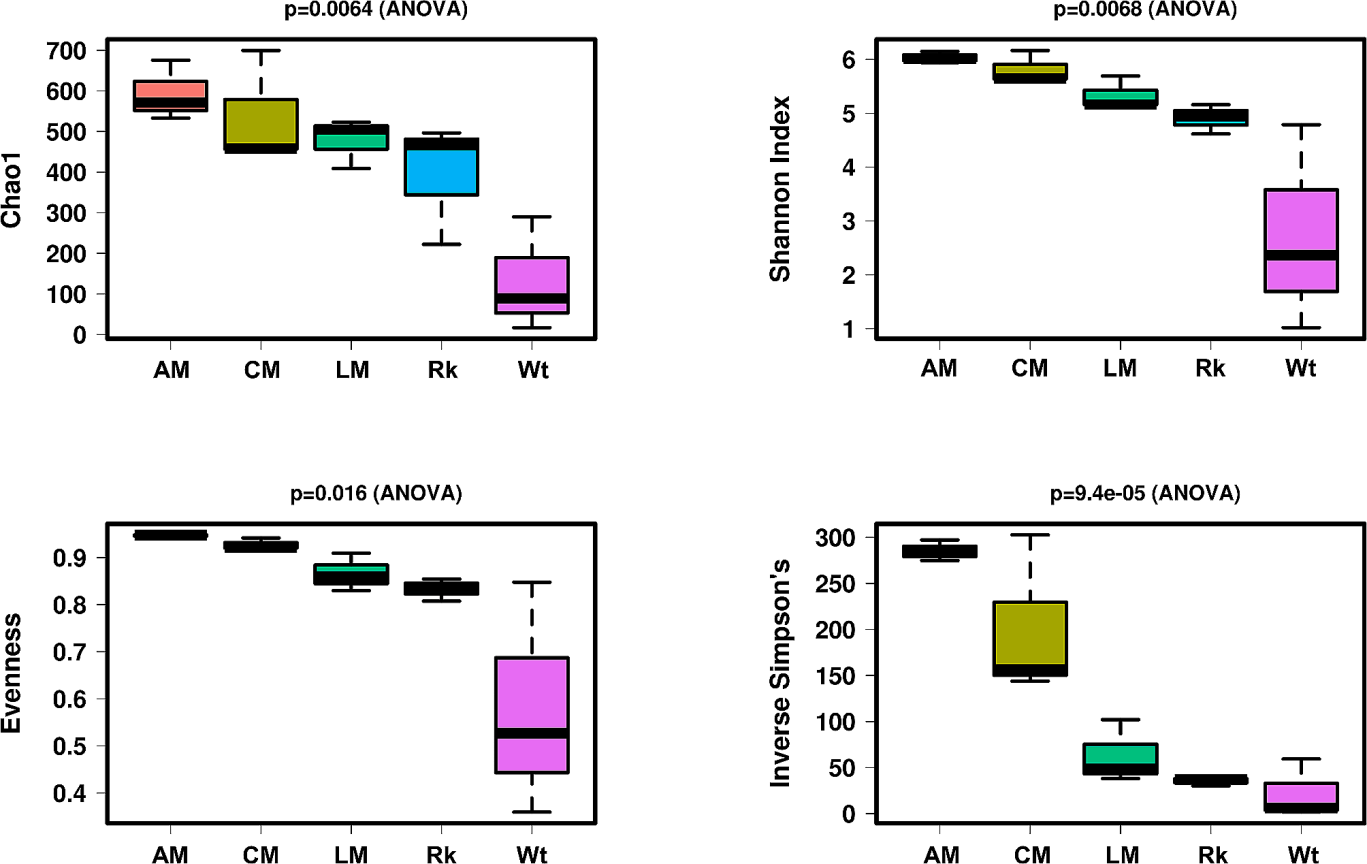
Box plots showing alpha diversity indexes (Shannon, Inverse Simpson’s, Chao1, and Evenness) variation across samples on rarefied data

**Fig. 5:**
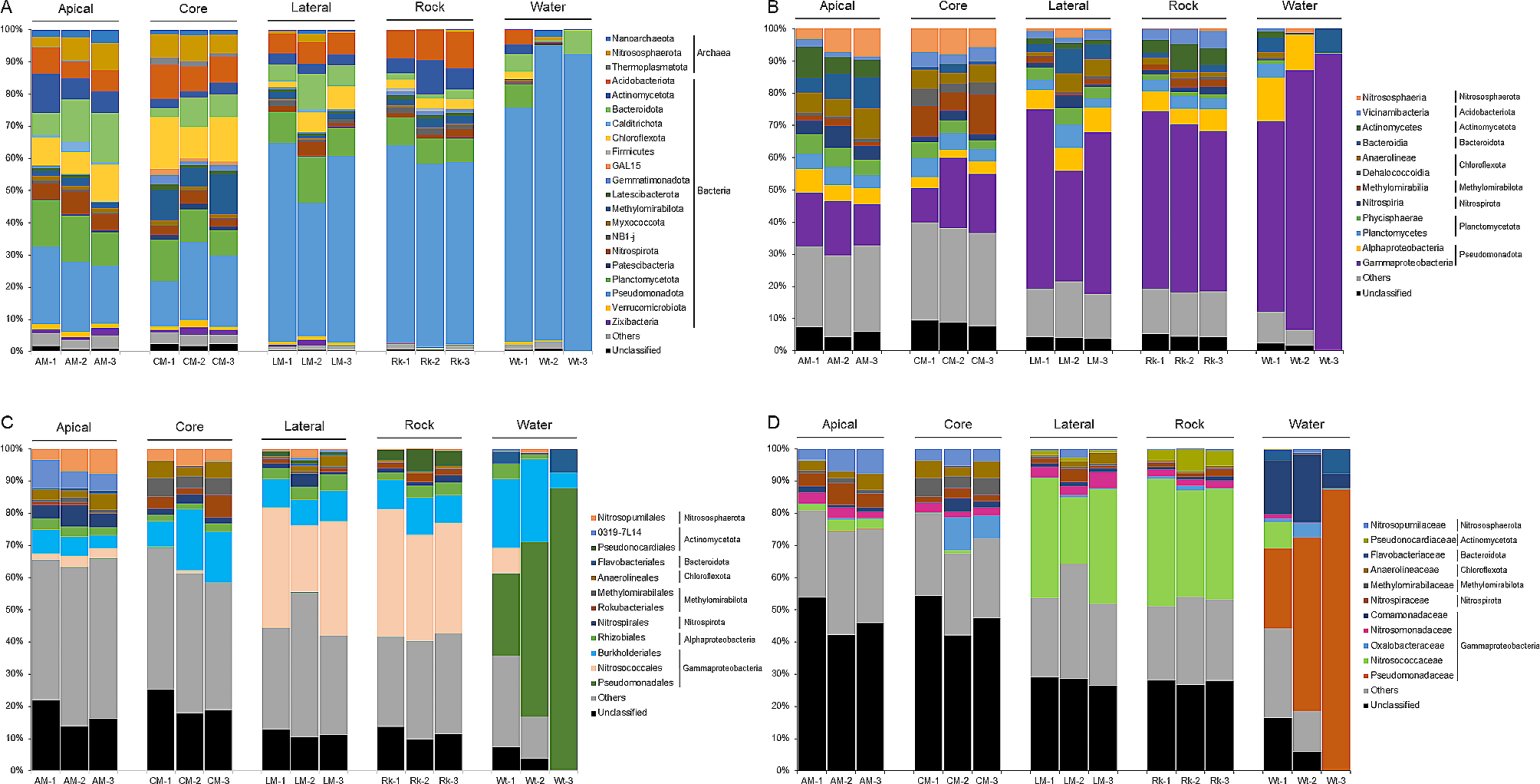
Relative abundance of prokaryotic phyla **(A)**, classes **(B)**, orders **(C)**, and families **(D)** in each sample representative of the apical (AM-1, -2, -3), core (CM-1, -2, -3), lateral parts (LM-1, -2, -3) of the three different moonmilk structures, the bedrock (Rk-1, -2, -3), and the waters (Wt-1, -2, -3). Prokaryotic phyla with abundances > 1% **(A)**, and prokaryotic classes **(B)**, orders **(C)**, and families **(D)** with abundances > 5% in at least one sample are displayed in the histograms. “Others” include low abundant taxa (phyla < 1%; classes, orders, and families < 5%)

The alpha diversities were estimated through Chao1 index, which measures richness, and the Inverse Simpson’s, Shannon, and Evenness indexes, which combine both richness and evenness. As a result, these diversity indexes showed a common trend that followed the sequence of samples: apical > core > lateral > rock > water (Fig. 4). The apical and core samples of moonmilk had the highest microbial diversity and richness followed by the lateral part of the moonmilk together with the bedrock samples. The water samples showed the lowest values of diversity and evenness among all the samples considered. This diversity trend could be observed already at high taxonomy levels (phyla, classes, and orders) (Fig. 5**)**. At phylum level, the microbial communities in the moonmilk core were constituted by 17–18 phyla with abundance > 1%, followed by the apical part with 13–14 abundant phyla and the lateral part with 9–11 phyla. In the case of bedrock, 11–12 phyla had abundance > 1%, although only *Pseudomonadota* was > 10%. Water samples showed a higher heterogeneity with a number of phyla ranging between 2 and 6.

### Beta-diversity and community structure correlation with environmental parameters

**Fig. 6:**
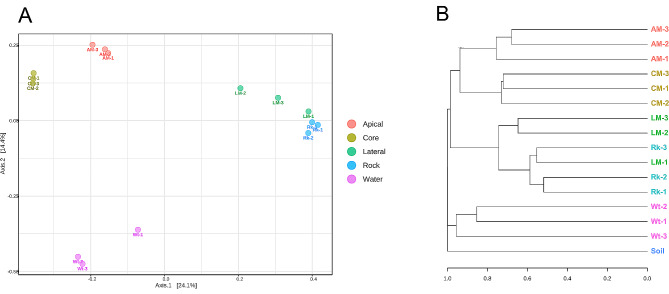
Beta-diversity analysis of the microbiota present in the diverse moonmilk parts, bedrock, and water bodies. **(A)** Principal Coordinates Analysis (PCoA) based on Bray-Curtis distance matrix and PERMANOVA statistical method of the microbial communities at ASV level. **(B)** Bray-Curtis based dendrogram of the different Grotta Nera samples at ASV level

Bray-Curtis-based analysis showed that the samples grouped depending on the type of matrix and moonmilk parts (Fig. 6**).** Furthermore, moonmilk core samples grouped with apical samples, while lateral samples clustered with the bedrock. This indicates that the microbial communities inhabiting the lateral part of the moonmilk are more similar to those present in the bedrock compared to the other two parts of the speleothem structure (i.e., core and apical parts). On the other hand, the water samples collected from the cave clustered apart from all the moonmilk parts and the bedrock samples (Fig. 6). These results indicate the presence of completely different microbial populations in the water bodies from the speleothem and bedrock samples that were collected from the cave ceiling. Furthermore, water samples showed Bray Curtis dissimilarity > 0.85 indicating a much lower level of relatedness and therefore higher heterogeneity of the microbial populations present in the water bodies. This could be due to a much higher diversification of environmental inputs that can influence the water ponds that are located on the cave ground floor and collect moonmilk dripping. The soil sample that was analyzed as a control did not cluster with any cave sample in the beta-diversity analysis, indicating that the microbial communities inhabiting the cave are distinct from those present in the soil above the cave.

The microbial community structure was further correlated to the environmental parameters listed in Table 1, i.*e.*, the cave site of the sampling (nearest to the cave entrance, in the middle of the cave, in the deepest zones of the cave), the type of matrix considering the moonmilk all together or divided into parts (i.e. moonmilk, rock, water in the first case, and apical, lateral and core part of the moonmilk, rock, water in the second case), water content, pH value. Principal Coordinates Analysis (PCoA) based on PERMANOVA statistical analysis (Fig. 6, **Additional file 1: Fig. S4**) revealed that the microbiota composition was correlated with the type of matrix when the moonmilk samples were considered all together or subgrouped based on the three parts of the speleothem (*p* = 0.003 and *p* = 0.001, respectively). On the other hand, there was no correlation between microbiota and the cave locations (*p* = 0.981), indicating that the moonmilk is characterized by specific microbial communities that are conserved among different moonmilk speleothems independently of their relative proximity to the cave entrance or the cave depth. This result can also be associated to the presence of similar environmental conditions in the second room where all sampling was carried out.

### The microbiota associated with bedrock, water and each moonmilk niche

**Fig. 7:**
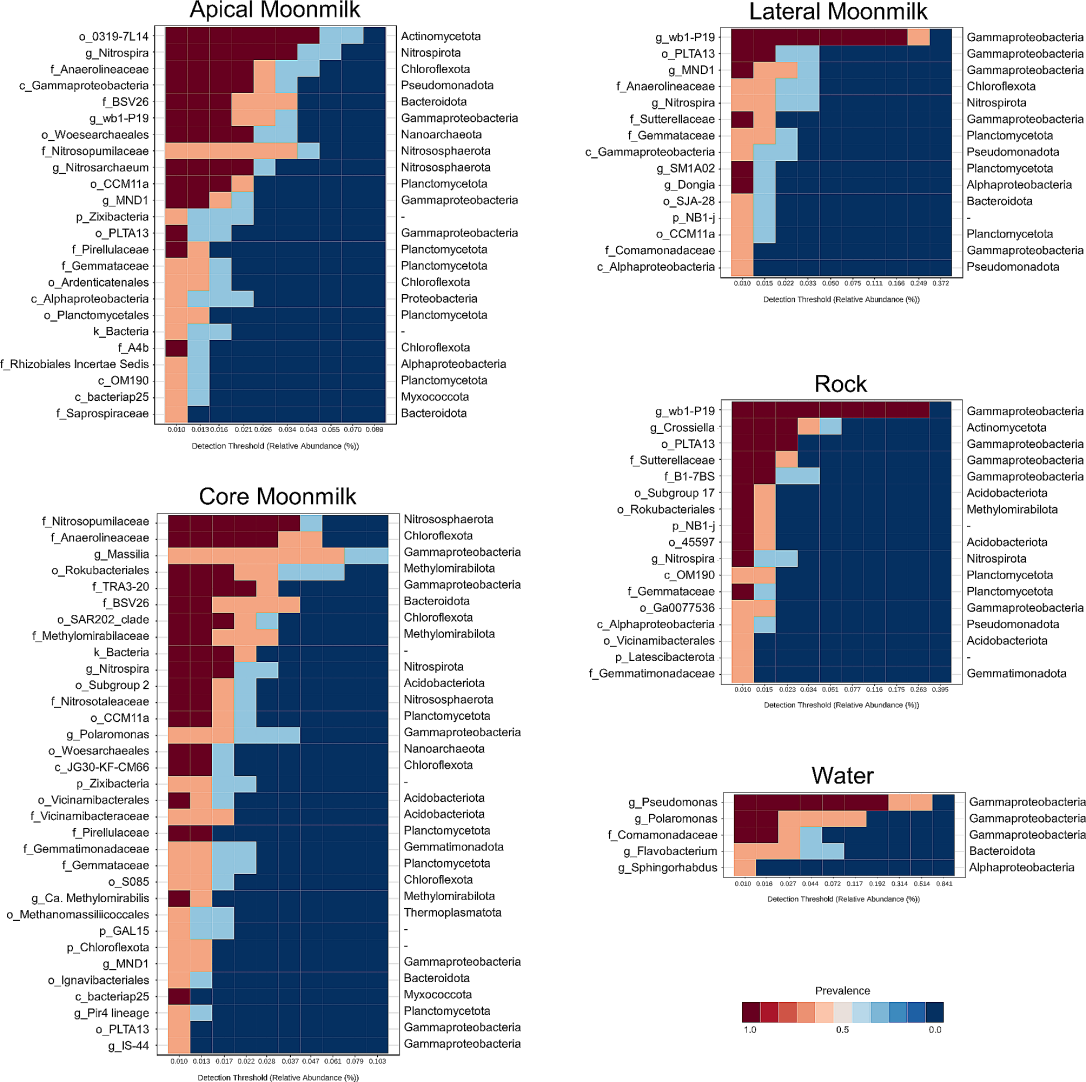
Core microbiome analysis of each sample group/cave niche with prevalence > 50% and relative abundance 0.01 at the highest classified taxonomy level. The phylum or class of each taxon is reported on the right of each graph

In order to define the microbiota distinguishing the bedrock from the moonmilk speleothem, we analysed the core microbiome in each sample group (Fig. 7) and performed Lefse analysis (**Additional file 1: Fig. S5**) by comparing all the microbial communities found in the different moonmilk niches with the bedrock (i.e., unmodified rock substrate on the cave ceiling) and the water (i.e., water sampled from water bodies on the floor collecting water dripping from the moonmilk). As a result, moonmilk was the only sample group that was characterized by archaeal taxa that, conversely, were present only in traces in the cave bedrock and waters (**Additional file 1: Fig. S6)**. These moonmilk-associated archaeal members were enriched in the core and apical part of the speleothem and belonged to the phyla *Nanoarchaeota* (*Woesearchaeales* order), *Nitrososphaerota* (*Nitrosopumilaceae* family) and, at lower abundance, *Thermoplasmatota (*of *Methanomassiliicoccales* order). In particular, the apical part of moonmilk was enriched by the genus *Nitrosarchaeum* that includes aerobic ammonia-oxidizing members [49]. The core part of moonmilk also showed a specific enrichment of the archaeal family *Nitrosotaleaceae* (*Nitrosospheria* class). The main bacterial phyla associated with moonmilk were *Bacteroidota*, *Chloroflexota*, *Nitrospirota*, *Planctomycetota*, *Methylomirabilota*, and, at lower abundance, *Verrucomicrobiota*, *Zixibacteria*, and *Fibrobacterota*. At lower taxonomy levels, all three moonmilk samples shared a high abundance of the genus MND1 of the *Nitrosomonadaceae* family, and the genus *Nitrospira* of the *Nitrospirota* phylum. They also showed similar abundance (> 1%) of the families *Phycisphaeraceae* and *Gemmataceae* of *Planctomycetota*. Other moonmilk-associated bacterial taxa were highly abundant in the core and apical parts and presented < 1% in the lateral part. These taxa comprised the genus BSV26 of *Bacteroidota*, the family *Anaerolineaceae* of *Chloroflexota*, the family *Methylomirabilaceae* of *Methylomirabilota*, and the order CCM11a of *Planctomycetota*. Finally, some bacterial lineages characterizing the moonmilk were highly abundant only in the core sample; these were the Subgroup 2 of *Acidobacteriota* and the *Ca.* Methylomirabilis of *Methylomirabilota*, the family TRA3-20 of *Planctomycetota*, the JG30-KF-CM66 class and SAR202 clade of *Chloroflexota*, the genus *Massilia* of *Oxalobacteraceae* family (*Burkholderiales* order). Only the apical part showed an enrichment of the order 0319-7L14 belonging to the *Actinomycetota* phylum.

The main phyla characterizing the bedrock were the *Pseudomonadota*, *Actinomycetota*, *Acidobacteriota*, *Gemmatimonadota*, the NB1-j, and *Latescibaterota* phyla. At low taxonomic levels, the main *Pseudomonadota* class was *Gammaproteobacteria* (49–55%) followed by *Alphaproteobacteria* (4.8–6.7%) of *Rhizobiales* order. *Gammaproteobacteria* were mostly constituted by members of the wb1-P19 genus (32–39%, *Nitrosococcales* order) and unclassified members of the *Sutterellaceae* and B1-7BS families (*Burkholderiales* order) and of the order PLTA13. Other abundant taxa in the bedrock belonged to the *Acidobacteriota* orders Subgroup 17, *Vicinamibacterales*, 11–24 and 45,597, the *Planctomycetota* families *Gemmatimonadaceae* and *Pirulellaceae*, and the *Actinomycetota* genus *Crossiella* (*Pseudonocardiales* order). Unclassified members of the phylum NB1-j, *Rokubacterales* order (*Chloroflexota* phylum) and *Nitrospira* genus (*Nitrospirota* phylum) were > 1% in all the bedrock samples under analysis. Some of the highly abundant taxa in the bedrock samples were also enriched in the lateral part of the moonmilk and present in the apical part, these mainly belonged to the genus wb1-P19, the family *Sutterellaceae* and the order PLTA13.

In the case of the water samples, there was a higher composition heterogeneity compared to the other matrixes/niches already at the phylum level. The microbial communities in the water bodies were constituted by 72–92% of *Gammaproteobacteria*, mostly of *Pseudomonadales* and *Burkholderiales* orders of *Pseudomonas* and *Polaromonas* genera and unclassified genus of *Comamonadaceae* family (Fig. 5). *Pseudomonadota* in water samples also comprised members of *Alphaproteobacteria* class although at much lower abundance (< 15%), they mostly belonged to *Sphingorhabdus* genus. After *Pseudomonadota*, the most abundant phylum in water was *Bacteroidota*, mostly composed by members of *Flavobacteriales* order and *Flavobacterium* genus.

### Phylogeny of dominant ASVs and affiliation with reference microbial strains

**Fig. 8:**
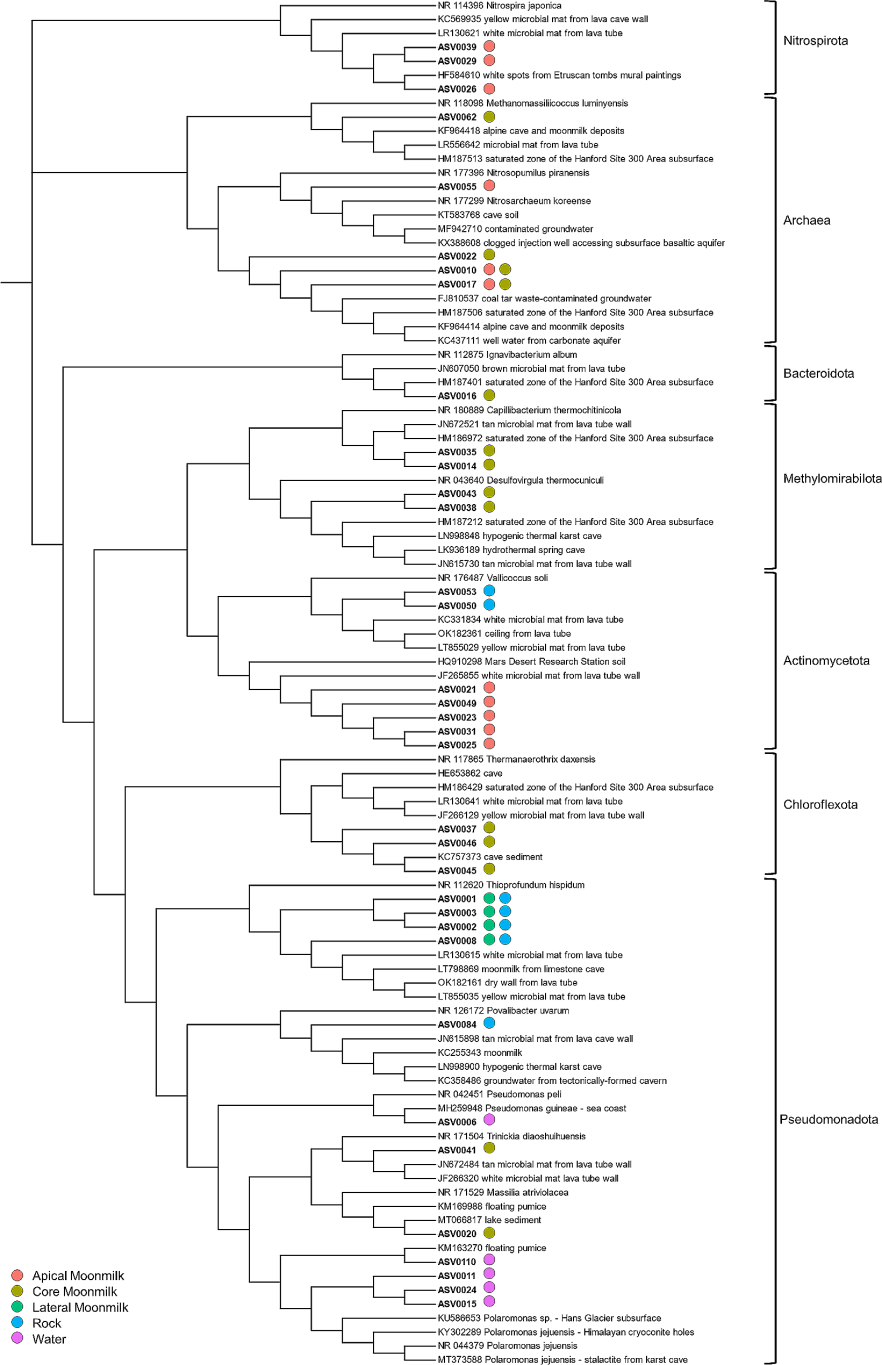
Neighbor-joining phylogenetic tree including the most abundant ASVs in the Grotta Nera samples and the most closely related sequences retrieved from the database

In total, 35 ASVs were highly abundant in each matrix (i.e., ASVs that were present in all the three samples representative of each group and > 1% in at least one of these samples). These ASVs were reported on a phylogenetic tree together with their closest relatives including the most affiliated strains that are taxonomically classified in the NCBI database (Fig. 8, **Additional file 1: Fig. S7**). Twenty-six ASVs out of 35 (74%) shared < 97% sequence identity with cultured prokaryotes. Among these, 17 ASVs that were enriched in the apical and core moonmilk niches, shared < 90% with cultured reference strains, indicating the high presence of undescribed microbial taxa associated with the moonmilk niches. In the apical moonmilk these ASVs were affiliated to the 0319-7L44 order (*Actinomycetota*) and their most correlated reference sequences were found in white microbial mats from lava tubes and in soils from the Mars Desert Research Station. In the core moonmilk these ASVs belonged to the BSV26 family (*Bacteroidota*), *Methylomirabilaceae* family (*Methylomirabilota*), *Rokubacterales* order (*Methylomirabilota*), *Methanomassiliicoccales* order (archaeal *Thermoplasmatota*) and *Anaerolineaceae* family (*Chloroflexota*), which were highly similar (> 99%) with clones retrieved from karst caves, lava tubes, moonmilk deposits, and a subsurface of a semi-arid contaminated area (Hanford Site) (Fig. 8). One additional ASV characterized the core moonmilk and was > 99% similar to clones found on floating pumice on a lake and with a cultivated strain of *Massilia atriviolacea* isolated from soil (Fig. 8, **Additional file 1: Fig. S7**).

The dominant ASVs in the lateral moonmilk corresponded with the ASVs dominant in the bedrock and were affiliated to the wb1-P19 genus, with their closest relatives retrieved from yellow/white microbial mats in lava tubes and from a moonmilk in a limestone cave (Fig. 8). Two additional bedrock-associated ASVs were affiliated with *Crossiella* genus and were similar to uncultured clones retrieved from colored microbial mats found in lava tubes.

The water samples were characterized by ASVs affiliated to *Polaromonas* and *Pseudomonas* genera that shared > 97% of identity with cultured representatives from the NCBI database. The *Polaromonas* ASVs were similar to clones retrieved from stalactites in a karst cave, Himalayan cryoconite holes, the Hans Glacier subsurface, and the volcanic Halla Mountain, while the *Pseudomonas* ASVs were similar to bacteria isolated from a seacoast and from a nitrifying inoculum and from a floating pumice (Fig. 8, **Additional file 1: Fig. S7**).

### Correlation between microbial diversity and molecular composition of preserved organic matter

**Fig. 9:**
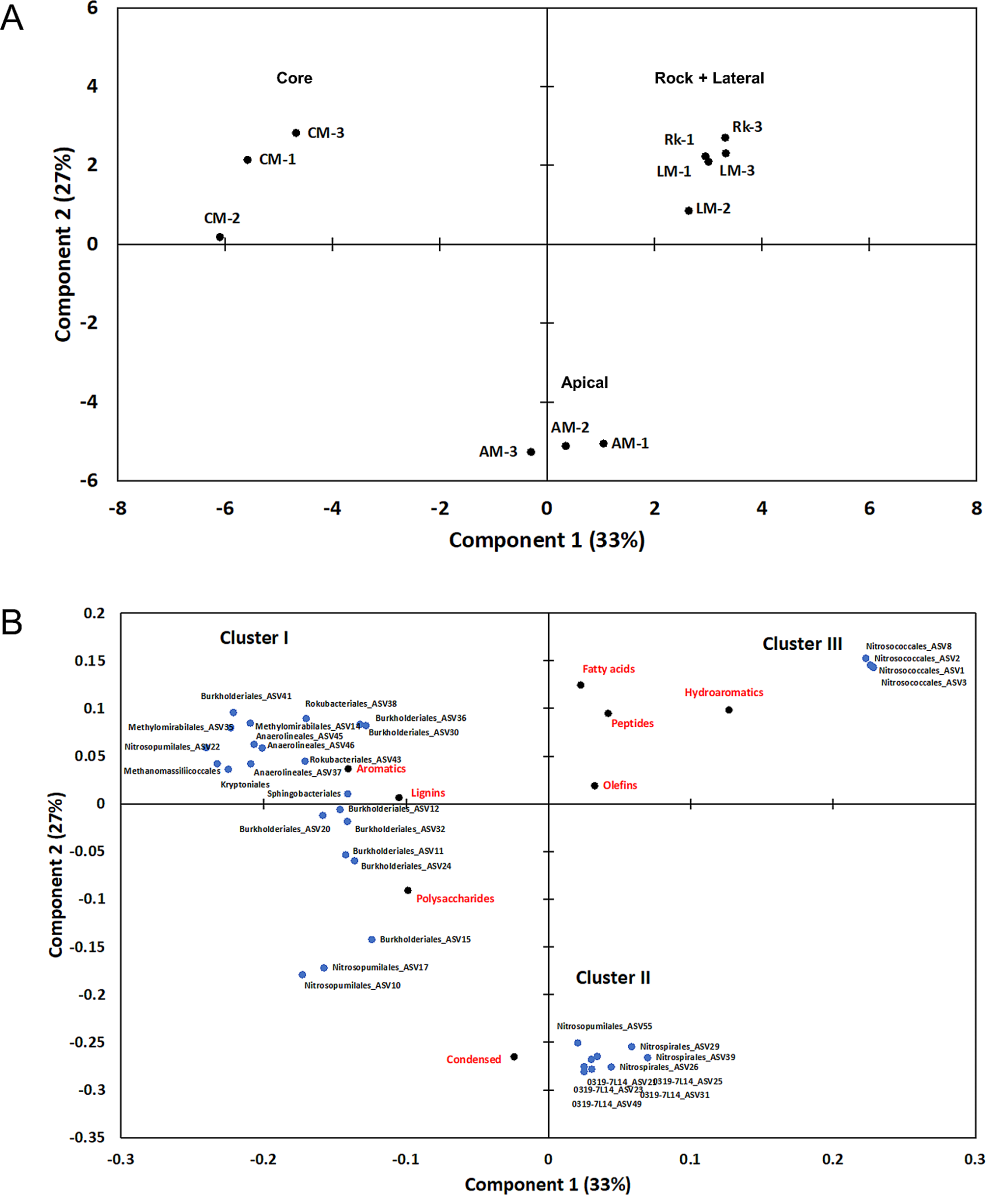
Principal components analysis (PCA) plots showing **(A)** the clustering of the Grotta Nera samples based on the molecular composition and **(B)** the correlation between the organic families (detected through analytical pyrolysis) and the most abundant ASVs present in each niche (considering the order taxonomy level)

The principal component analysis (PCA) of the organic families obtained by Py-GC/MS and the most abundant microbial taxa found in the speleothem samples are plotted in Fig. 9. The PCA explains 60% of the total variance using only the first two components (PC1: 33% and PC2: 27%) and defines three clusters (Fig. 9): (i) cluster I groups the samples collected from the core part of the moonmilk speleothems; (ii) cluster II is dominated by samples from the apical part, and (iii) cluster III comprises the samples collected from the lateral part and the bedrock. The PCA also shows the existence of significant differences among the different parts of the moonmilk, but there are no significant differences among the locations of moonmilk in the cave. The loading PCA plot (Fig. 9) shows that each cluster is dominated by specific organic families and microbial taxa. The moonmilk samples from the core are mainly composed of aromatic, polysaccharide and lignin compounds, showing the highest molecular diversity in terms of OM. This diversity is also observed in the microbial data, being the sample depicting the highest microbial diversity. In contrast, cluster II (the apical part of moonmilk) is highly influenced by condensed compounds (recalcitrant molecules like polycyclic aromatic hydrocarbons). This cluster is dominated by bacteria l orders belonging to the phyla *Nitrospirota* (*Nitrospirales*), *Actinomycetota* (*0319-7L14*), and *Nitrososphaerota* (*Nitrosopumilales*). Finally, the OM preserved in the lateral part of the moonmilk and bedrock samples are composed of peptides, fatty acids, hydroaromatics, and olefin compounds. The cluster III only shows a correlation with one bacterial order belonging to the *Pseudomonadota* phylum (*Nitrosococcales*).

## Discussion

This work aimed to provide the first systemic analysis of different parts/niches of the huge moonmilk structures present in Grotta Nera, a protected limestone cave in the Majella Park (Abruzzo, Italy). Moonmilk is a rather common type of carbonate deposit (speleothem) found in caves and its formation is thought to involve microbial activities and interactions that are still not fully elucidated [1, 11, 16, 20, 23]. These moonmilk structures in Grotta Nera are unique in terms of numerosity and dimension. Because of the huge size and shape, these speleothems can be divided into three main parts/niches represented by the apical, the lateral, and the core parts. These three niches have different macroscopic features, including the softness, the texture, the white color tuning (being the lateral part more greyish that the core and apical ones), and the water content. The microbiological analyses and molecular composition of the moonmilk samples in Grotta Nera showed that each moonmilk part/niche was characterized by specific microbial communities and organic matter compositions regardless of their position inside the cave. This can be due, on one hand, to the presence of rather stable values of pH (neutral), temperatures, and CO_2_ in the second room, where moonmilk deposits are abundant (and where the different moonmilk samples were collected), with only slight modifications among seasons. Carbon isotope analysis of the moonmilk niches/parts indicates a significant processing of the organic matter deriving from the aboveground soil, probably due to microbial activity. This fact has reflection in the microbiota thriving in Grotta Nera, which is strongly diverse from that colonizing the aboveground soil. Indeed, < 3% of the microbial species (ASVs) found in the cave were shared with the soil microbiota (**Additional file 1: Fig. S8**) and only a few dominant taxa in the soil were also highly abundant in Grotta Nera samples (**Additional file 1: Fig. S9**). These results indicate that the cave environment has evolved into an independent ecosystem associated with a specific microclimate and with specific microbial communities adapted to survive under oligotrophic conditions.

The organic matter (OM) composition analysis grouped the bedrock and the moonmilk lateral samples together and separated them from the moonmilk apical and core parts. Therefore, the lateral and bedrock samples showed a certain degree of similarity in the OM composition, while the other two parts of the moonmilk were distinct. The OM composition of the soil above the cave also showed similarities with the bedrock samples, indicating that the surface soil is the source of the organic matter recorded inside Grotta Nera. A main factor driving the OM flow is the water dripping into the cave that greatly influences the speleothem growth and chemistry and is affected by surface conditions like precipitation, changes in temperature, airflows, soil cover, and type of vegetation [50, 51]. In this regard, the main tree species present above the cave are beech (*Fagus sylvatica* L.), golden chain tree (*Laburnum anagyroides* Medik.), mountain ash (*Sorbus aucuparia* L.), hornbeam (*Carpinus betulus* L.), and mountain pine (*Pinus muga* Turra). Accordingly, the bedrock samples generally showed the highest diversity of molecular composition, with a relatively high proportion of fresh material (e.g., polysaccharides, olefins, hydroaromatics, and lignins), which may come from the aboveground vegetation through leaching processes [30, 31]. Vegetation biomarkers, such as lignin (methoxyphenols) and long-chain *n*-alkanes, were also identified in the speleothems, particularly in the core part, suggesting that the Grotta Nera moonmilk are archives of environmental changes occurred in the area above the cave [32]. It is noteworthy that refractory molecules, such as polyaromatic hydrocarbons and condensed chemicals, were detected in both the core and apical parts of the speleothems and might have derived from ancient fire events on the surface. The existence of these very resistant and hydrophobic compounds (condensed) in the apical section might be due to the assimilation of soot particles from fossil fuel burning via leaching processes [31, 52, 53], although there is no recent record of wildfire events within the study area. In several cases, the OM preserved in the speleothems could be associated with microbial presence and activity, in relation with the detection of peptide-like (diketopyperazines) and lipid-like compounds (low molecular weight alkyl compounds, < C_20_) (**Additional file 1: Table S2**) [48, 54]. While the diketopyperazines were mainly detected in the lateral region of the speleothems and bedrock, various low molecular weight alkyl compounds were enriched in the different niches under analysis. The similarity of the OM composition between the bedrock and lateral samples, including the OM compounds that can be ascribed to microbial biomass and activity, could partly explain the similarity detected between the microbial communities colonizing these two niches (in terms of alpha- and beta-diversity as well as microbial composition) and their differences from the core and apical parts.

In addition to the OM composition, the various Grotta Nera parts/niches showed different water content that is known to be a main driving factor of biodiversity in subterranean environments [5]. In particular, the higher abundance of prokaryotic cells, biodiversity, and richness observed in the moonmilk niches might be related with the higher values of water content found in these niches compared to the bedrock and with the possible increased amount of solubilized nutrients due to advanced biomineralization processes [55]. Furthermore, specific prokaryotic taxa (both bacteria and archaea) were specifically enriched in the speleothems indicating that the development of the Grotta Nera moonmilk involved a complex evolution process that led to niche/part diversification and specific microbial community evolution/selection. Moonmilk-associated taxa were *Bacteria* and *Archaea* frequently unclassified at low taxonomy levels (family and genus) and affiliated with taxa known to be anaerobic. These taxa were highly abundant in the core and apical parts, while their abundance decreased moving to the lateral zone and were undetectable in the bedrock and water samples. This suggests that the microbial diversification in Grotta Nera follows a depth- and oxygen-dependent stratification like previously suggested for archaeal taxa in alpine moonmilk [56]. These results differ from a previous work on moonmilk from Pindal Cave [57], which did not find a strong microbial diversification between the speleothem and the unmodified bedrock, probably related to the more limited dimension and 3D structure of the moonmilk in this other cave.

**Fig. 10:**
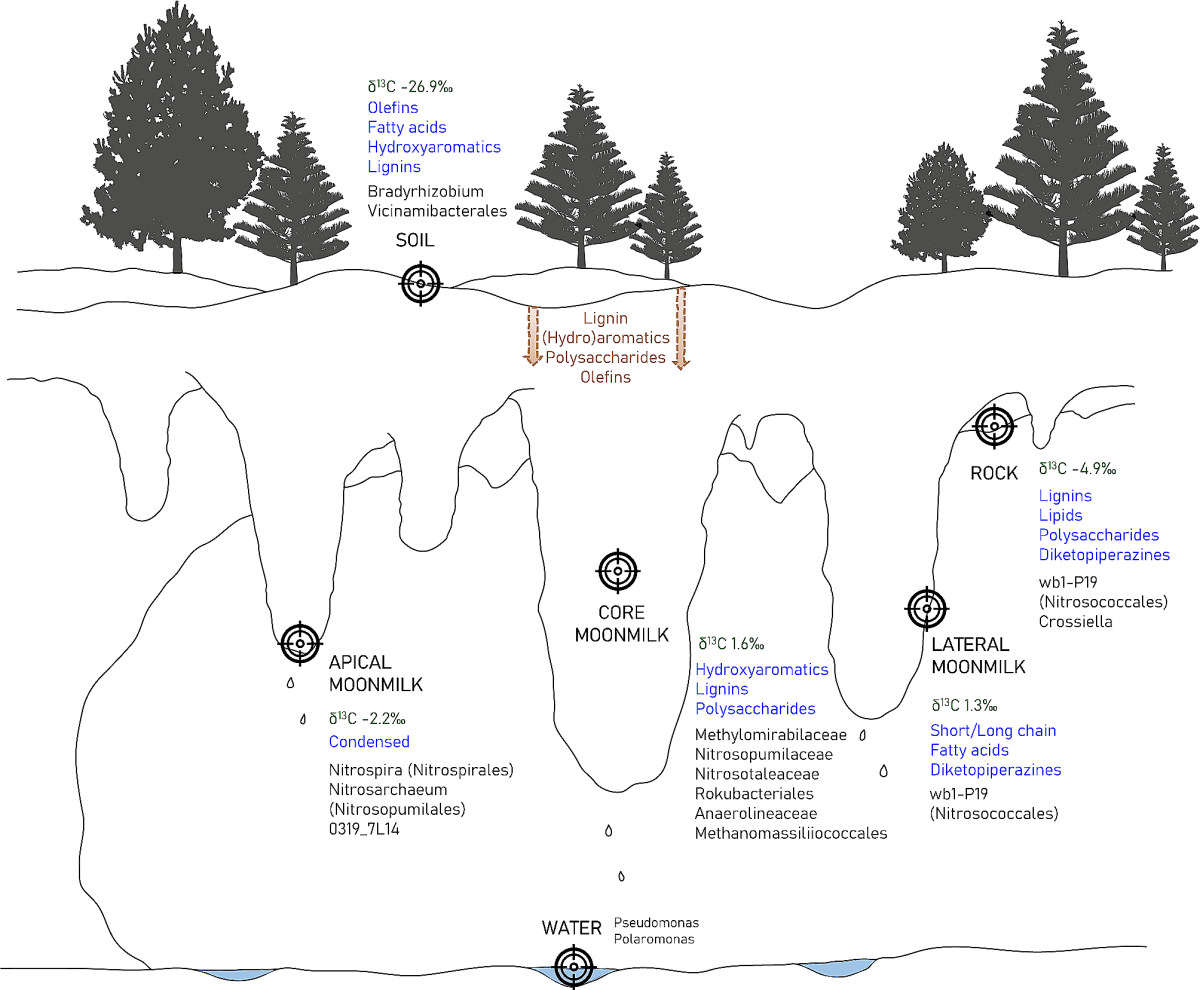
Conceptual model of Grotta Nera showing the different niches associated to the main isotopic data (in green), molecular organic composition (in blue) and prokaryotic taxa (in black). Brown arrows indicate the possible transfer/movement of organic matter from the soil above the cave

Some of the microbial taxa enriched in each moonmilk niche could be correlated with OM composition suggesting that the OM preserved in the speleothems might contribute to the shaping of the microbiota colonizing the different parts of the moonmilk (Fig. 10**)**. The orders *Nitrospirales* (*Nitrospira* genus), 0319-7L14 (of *Actinobacteriota* phylum), and *Nitrosopumilales* (*Nitrosarchaeum* genus), that are dominant in the apical part, were strongly correlated with condensed organic compounds, probably due to their ability to degrade these recalcitrant compounds [58, 59]. Diverse microbial orders abundant in the core part were correlated with aromatic, lignin, and polysaccharides and some of them, i.e., *Burkholderiales* (mainly of *Massilia* genus), *Kryptoniales* (family BSV26), *Anaerolineales*, and *Rokubacteriales*, are known to degrade aromatic and lignin compounds in different ecosystems (e.g., soil, water, and sediments) [39, 60–62]. Finally, the *Nitrosococcales* (genus wb1-P19) present in the lateral part and bedrock are correlated with fresh organic matter (peptides, fatty acids, hydroaromatics, and lipids), which are typically associated with active microbial metabolism.

Microbial taxa enriched in moonmilk speleothems could be also associated with the metabolism of C_1_ gases (CO_2_ and CH_4_) and inorganic nitrogen that were also previously detected in biofilms from other oligotrophic caves [56, 63–65]. Among these taxa, we found members of the families *Nitrosopumilaceae, Nitrosomonadaceae*, and *Nitrosotaleceae* and genera *Nitrosoarchaeum* and *Nitrospira*, which are ammonia and/or nitrite oxidizers, some of them also being able to fix CO_2_. We also found members of *Methylomirabilaceae* and *Candidatus* Methylomirabilis, which are known as denitrifying methanotrophs, and the archaeal order *Methanomassiliicoccales* that includes methanogens. This result suggests a possible role of methane and inorganic nitrogen metabolisms and/or associated taxa in moonmilk development process (involving carbonate dissolution and re-precipitation). Accordingly, aerobic and anaerobic oxidation of methane and nitrate reduction were previously found to influence redox conditions and lead to carbonate dissolution and precipitation in diverse environments [10]. Moreover, low concentrations of oxygen and methane were reported to trigger exopolysaccharide (EPS) production in methanotrophs [66, 67] that can function as nucleation sites for carbonate precipitation.

Among the microbial taxa highly abundant in the Grotta Nera bedrock, the genera wb1-P19 and *Crossiella* were absent or with low abundance (< 1%) in the apical and core parts of the moonmilk. The only wb1-P19 was also significantly present in the lateral part of the moonmilk. Members of wb1-P19 are phylogenetically affiliated with nitrite oxidizing bacteria with chemoautotrophic activities and aerobic respiration. This taxon has been frequently found in caves associated with microbial mats, moonmilk, and weathered rock [7, 19, 68]. In particular, probably in association with the aerobic metabolism, wb1-P19 was found to be predominant in the surface layers of both the bare substrate and the moonmilk samples in Pindal Cave, while its abundance decreased below 5% in the deepest layers that were collected and analyzed [57]. On the other hand, in Pindal Cave moonmilk study, *Crossiella* species were previously found to be dominant not only in the bare cave substrate but also in not structured and flat moonmilk that might correspond to stages of speleothem formation prior to those characterizing Grotta Nera moonmilk. Therefore, the differential presence of *Crossiella* in the two types of moonmilk might be related to the role of this genus in the initial development stages of the speleothem. In line with this, *Crossiella* strains were previously indicated to be able to precipitate carbonate and phosphate minerals [69].

## Conclusions

In this work, we have assessed the microbiology and organic molecules composition of the huge moonmilk speleothems inside Grotta Nera, which represent unique ecosystems to analyze the microbial communities colonizing and interacting with the carbonatic host rocks. Our analyses revealed that moonmilk deposits are divided into four main niches/parts, i.e., bedrock, moonmilk lateral, apical, and core which, based on organic matter and microbiology analyses, can be clustered into two main groups, one group including the core and apical niches of the speleothem and the other including the bedrock and the lateral niches. These niches are featured by specific microbial communities and the enrichment of specific taxa mostly follow oxygen and water gradients (along the moonmilk depth) as well as organic matter composition. Analytical pyrolysis identified the molecular compounds that were found to originate from microbial activities and/or from soil and vegetation above the cave (Fig. 10). The high microbial abundance and diversity in the moonmilk core and apical parts, both greater than in the other niches of the cave (bedrock and water ponds), suggest the presence of complex microbial activities and interactions related with rock mineral modifications. The enrichment of specific chemolithotrophic microorganisms was also observed suggesting that inorganic nitrogen and methane together with complex polymers from the vegetation above the cave function as driving factors for microbial community selection and carbonate rock biomineralization. Future studies will delve into the mechanisms leading to the development of the moonmilk speleothems in Grotta Nera by carrying out comparative metagenomic analyses and functional studies of the microbial communities colonizing the different moonmilk niches and the unmodified bedrock.

### Electronic supplementary material

Below is the link to the electronic supplementary material.

Additional File 1: **Table S1** Carbon isotope signatures (𝛿^13^C) of moonmilk and soil samples from the Grotta Nera cave (mean ± standard deviation in ‰, VPDB). **Table S2**. *n*-Alkanes, fatty acids, and triterpene compounds found in the moonmilk and bedrock samples. **Table S3**. Diketopiperazine compounds found in moonmilk and bedrock samples. **Fig. S1.** Evolution of the air temperatures present inside and outside Grotta Nera monitored over one year (from June 2019 to June 2020). The CO_2_ mean values detected during each season are also indicated. **Fig. S2.** Absolute quantification of the prokaryotic cell biomass present in each cave niche obtained through qPCR targeting specific regions of bacterial and archaeal 16 S rRNA gene. The abundance is expressed as number of copies of 16 S rRNA per gram or milliliter of sample. **Fig. S3.** Rarefaction curves of the Illumina sequencing data obtained from the fifteen cave samples. **Fig. S4.** Correlation between the Grotta Nera samples and different environmental parameters. Principal Coordinates Analysis (PCoA) based on Bray-Curtis distance matrix and PERMANOVA statistical method of the microbial communities at ASV level. The environmental parameters considered are the type of matrix/sample dividing the moonmilk into the core, apical, and lateral parts (A) or considering them together as moonmilk (B), and the cave location (C). **Fig. S5.** Cladograms showing the differences between moonmilk, bedrock, and waters samples in terms of microbial taxa according to LEfSe analysis with a LDA threshold of 3.5 and multiclass analysis strategy one-against-all. Taxa with significant differences are highlighted by colored circles and shadings. **Fig. S6.** Heatmap showing the abundance of the dominant prokaryotic taxa (with abundance > 1% in each sample group) detected in the Grotta Nera niches. **Fig. S7.** Heatmap showing the abundance and taxonomy affiliation of the dominant ASVs in Grotta Nera samples. The Best Blast classified Hit (BBcH) from NCBI, the identity percentage, the accession number, and the isolation source retrieved from NCBI 16 S rRNA sequences database are also reported. **Fig. S8**. (A) Venn diagram illustrating the ASVs shared between the moonmilk, rock, water, and soil samples. The ASVs shared between the soil and all the other samples are depicted in panel (B) together with their abundance (% of the total community) in each sample and the taxonomy. **Fig. S9.** Heatmap showing the distribution of the soil dominant taxa (> 1% in the soil) over the Grotta Nera samples.

## Data Availability

Sequence data that support the findings of this study have been deposited in the European Nucleotide Archive with the primary accession code PRJNA1052665.
