# Supplementary material for: The microbiota characterizing huge carbonatic moonmilk structures and its correlation with preserved organic matter
Source: Environ Microbiome. 2024 Apr 24;19:25. doi: 10.1186/s40793-024-00562-9 (PMC11040949; doi:10.1186/s40793-024-00562-9)
Supplement: Supplementary file 1 — Additional File 1: Table S1 Carbon isotope signatures (𝛿13C) of moonmilk and soil samples from the Grotta Nera cave (mean ± standard deviation in ‰, VPDB). Table S2. n-Alkanes, fatty acids, and triterpene compounds found in the moonmilk and bedrock samples. Table S3. Diketopiperazine compounds found in moonmilk and bedrock samples. Fig. S1. Evolution of the air temperatures present inside and outside Grotta Nera monitored over one year (from June 2019 to June 2020). The CO2 mean values detected during each season are also indicated. Fig. S2. Absolute quantification of the prokaryotic cell biomass present in each cave niche obtained through qPCR targeting specific regions of bacterial and archaeal 16 S rRNA gene. The abundance is expressed as number of copies of 16 S rRNA per gram or milliliter of sample. Fig. S3. Rarefaction curves of the Illumina sequencing data obtained from the fifteen cave samples. Fig. S4. Correlation between the Grotta Nera samples and different environmental parameters. Principal Coordinates Analysis (PCoA) based on Bray-Curtis distance matrix and PERMANOVA statistical method of the microbial communities at ASV level. The environmental parameters considered are the type of matrix/sample dividing the moonmilk into the core, apical, and lateral parts (A) or considering them together as moonmilk (B), and the cave location (C). Fig. S5. Cladograms showing the differences between moonmilk, bedrock, and waters samples in terms of microbial taxa according to LEfSe analysis with a LDA threshold of 3.5 and multiclass analysis strategy one-against-all. Taxa with significant differences are highlighted by colored circles and shadings. Fig. S6. Heatmap showing the abundance of the dominant prokaryotic taxa (with abundance > 1% in each sample group) detected in the Grotta Nera niches. Fig. S7. Heatmap showing the abundance and taxonomy affiliation of the dominant ASVs in Grotta Nera samples. The Best Blast classified Hit (BBcH) from NCBI, the identity per [file 40793_2024_562_MOESM1_ESM.docx]

**Supplementary material**

**Table S1.** Carbon isotope signatures (𝛿^13^C) of moonmilk and soil samples from the Grotta Nera cave (mean ± standard deviation in ‰, VPDB).

| Sampling site | Sample code | Location | 𝛿^13^C (‰, VPDB) |
| --- | --- | --- | --- |
| Innermost zone of the cave | AM-1 | Apical Moonmilk | -1.3 ± 0.4 |
|  | LM-1 | Lateral Moonmilk | 1.4 ± 0.0 |
|  | CM-1 | Core Moonmilk | 1.3 ± 0.1 |
|  | Rk-1 | Bedrock | -2.4 ± 0.2 |
| Middle zone of the cave | AM-2 | Apical Moonmilk | -2.0 ± 0.1 |
|  | LM-2 | Lateral Moonmilk | 2.2 ± 0.1 |
|  | CM-2 | Core Moonmilk | 1.6 ± 0.2 |
|  | Rk-2 | Bedrock | n.d.^a^ |
| Proximal to cave entrance | AM-3 | Apical Moonmilk | -3.2 ± 1.3 |
|  | LM-3 | Lateral Moonmilk | 0.4 ± 0.1 |
|  | CM-3 | Core Moonmilk | 2.0 ± 0.0 |
|  | Rk-3 | Bedrock | -7.6 ± 0.9 |
| Soil above the cave | SOIL | Soil | -26.9 ± 0.2 |

^a^ n.d.: not determined

**Table S2**. *n*-Alkanes, fatty acids, and triterpene compounds found in the moonmilk and bedrock samples.

| **Rt (min)** | **Molecular compound*** | **AM-1** | **CM-1** | **LM-1** | **Rk-1** | **AM-2** | **CM-2** | **LM-2** | **AM-3** | **CM-3** | **LM-3** | **Rk-3** |
| --- | --- | --- | --- | --- | --- | --- | --- | --- | --- | --- | --- | --- |
| 6.7 | Alk-C_11_ | 1.78 | 3.83 | 4.38 | 16.11 | 1.72 | 1.39 | 4.18 | 3.87 | 0.85 | 5.32 | 20.01 |
| 7.9 | Alk-C_12_ | 9.32 | 10.72 | 11.70 | 4.93 | 10.22 | 8.04 | 16.32 | 9.80 | 5.70 | 15.04 | 7.93 |
| 8.7 | Alk-C_13_ | 18.93 | 17.95 | 21.62 | 8.02 | 19.41 | 15.31 | 15.09 | 16.75 | 10.43 | 16.30 | 9.03 |
| 9.5 | Alk-C_14_ | 7.51 | 13.98 | 4.67 | 6.55 | 12.30 | 8.56 | 7.34 | 8.98 | 7.87 | 7.08 | 4.06 |
| 10.4 | Alk-C_15_ | 7.98 | 10.96 | 7.30 | 4.38 | 10.53 | 9.70 | 9.69 | 11.14 | 3.15 | 5.14 | 4.15 |
| 10.6 | FA C_16:0_ | 2.98 | 7.72 | 3.25 | 6.40 | 7.28 | 6.42 | 2.20 | 7.68 | 3.77 | 3.86 | 4.33 |
| 11.2 | Alk-C_16_ | 2.92 | 2.99 | 4.70 | 4.71 | 3.03 | 2.70 | 3.78 | 3.97 | 1.30 | 3.72 | 5.85 |
| 12.0 | Alk-C_17_ | 3.31 | 3.63 | 3.93 | 2.06 | 3.49 | 2.73 | 4.62 | 3.27 | 1.28 | 3.79 | 3.50 |
| 12.5 | FA C_18:0_ | 3.10 | 1.87 | 6.85 | 12.68 | 9.99 | 3.89 | 2.10 | 1.59 | 4.65 | 8.21 | 11.11 |
| 12.7 | Alk-C_18_ | 1.94 | 2.02 | 4.22 | 4.09 | 3.44 | 1.58 | 3.05 | 2.13 | 1.03 | 3.94 | 5.15 |
| 13.7 | Alk-C_19_ | 2.91 | 2.60 | 4.44 | 1.96 | 4.62 | 2.33 | 3.96 | 2.39 | 0.00 | 2.46 | 5.82 |
| 14.5 | Alk-C_20_ | 1.47 | 0.00 | 2.09 | 4.64 | 2.12 | 0.86 | 2.71 | 1.34 | 0.87 | 1.66 | 0.00 |
| 15.5 | Alk-C_21_ | 0.60 | 3.25 | 1.91 | 1.35 | 3.20 | 0.99 | 1.59 | 0.75 | 3.14 | 1.75 | 0.00 |
| 16.5 | Alk-C_22_ | 0.50 | 1.59 | 2.65 | 5.67 | 5.15 | 3.40 | 1.64 | 2.21 | 4.47 | 1.51 | 2.62 |
| 17.6 | Alk-C_23_ | 2.89 | 0.00 | 2.12 | 0.89 | 3.49 | 2.80 | 2.89 | 2.44 | 4.81 | 3.29 | 2.80 |
| 18.6 | Alk-C_24_ | 3.37 | 2.06 | 3.43 | 1.68 | 0.00 | 2.97 | 2.96 | 2.48 | 5.92 | 0.00 | 3.33 |
| 19.3 | Alk-C_25_ | 5.99 | 2.18 | 2.41 | 6.06 | 0.00 | 5.54 | 3.45 | 3.55 | 8.87 | 4.40 | 1.85 |
| 19.9 | Squalene | 3.86 | 2.49 | 2.65 | 0.94 | 0.00 | 4.16 | 3.56 | 4.54 | 8.20 | 4.31 | 2.97 |
| 20.6 | Alk-C_26_ | 6.26 | 3.08 | 1.87 | 1.84 | 0.00 | 6.99 | 2.25 | 4.55 | 9.57 | 0.00 | 1.61 |
| 21.3 | Alk-C_27_ | 5.51 | 0.00 | 1.86 | 2.40 | 0.00 | 3.25 | 2.51 | 4.39 | 6.22 | 3.73 | 2.03 |
| 22.0 | Alk-C_28_ | 3.25 | 2.79 | 1.09 | 0.74 | 0.00 | 3.90 | 1.73 | 0.00 | 5.58 | 4.49 | 1.14 |
| 22.5 | Alk-C_29_ | 3.62 | 4.29 | 0.84 | 1.90 | 0.00 | 2.49 | 2.39 | 2.17 | 2.32 | 0.00 | 0.70 |
|  | **ACL** | 16.53 | 14.49 | 14.33 | 13.66 | 12.85 | 15.76 | 15.47 | 14.69 | 17.28 | 13.84 | 13.05 |

*Alk, alkane; FA, fatty acid

** ACL, Average chain length

**Table S3**. Diketopiperazine compounds found in moonmilk and bedrock samples.

| Rt (min) | Diketopiperazine | Acronym | Ion M/Z* | MW** | AM-1 | CM-1 | LM-1 | Rk-1 | AM-2 | CM-2 | LM-2 | AM-3 | CM-3 | LM-3 | Rk-3 |
| --- | --- | --- | --- | --- | --- | --- | --- | --- | --- | --- | --- | --- | --- | --- | --- |
| 10.9 | Cyclo(hydroxyproline-hydroxyproline) | **Hyp-Hyp** | 65,93,94,130,**186** | 226 |  |  | X | X |  |  |  |  |  | X | X |
| 11.2 | Cyclo(proline-glycine) | **Pro-Gly** | 41,70,83,**111** | 154 |  |  |  | X |  |  |  |  |  |  | X |
| 11.8 | Cyclo(proline-valine) | **Pro-Val** | 41,**70**,72,125,154 | 196 |  |  |  | X |  |  |  |  |  |  | X |
| 12.4 | Cyclo(proline-leucine) | **Pro-Leu** | 44,70,86,125,**154** | 210 |  |  | X | X |  |  |  |  |  | X | X |
| 12.6 | Pyrrolo[1,2-α]pyrazine-1,4-dione, hexahydro-3-(2-methylpropyl) | **-** | 41,70,86,125,**154** | 210 |  |  | X | X |  |  |  |  |  | X | X |
| 12.7 | Cyclo(proline-proline) | **Pro-Pro** | 41,**70**,71 | 194 |  |  | X | X |  |  |  |  |  | X | X |

*Bold numbers represent the ion with the highest intensity.

**MW, Molecular weight


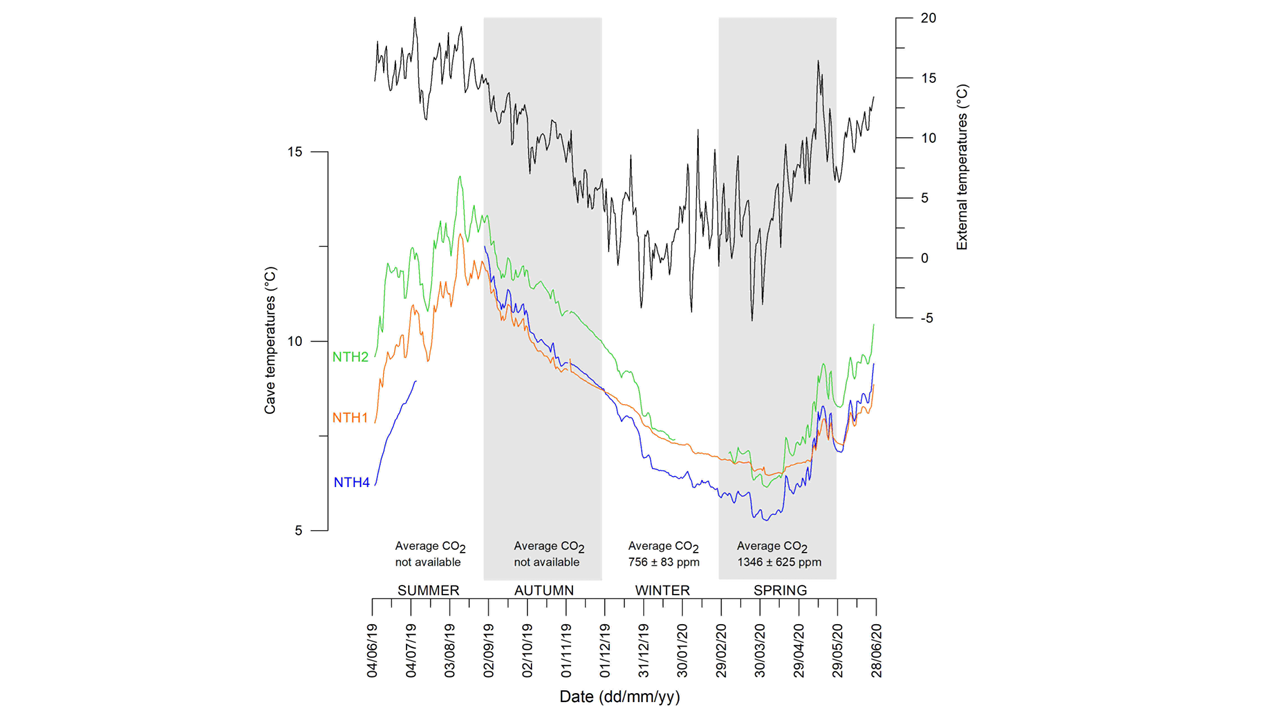


**Fig. S1.** Evolution of the air temperatures present inside and outside Grotta Nera that were monitored over one year (from June 2019 to June 2020). The CO_2_ mean values detected during each season are also indicated.


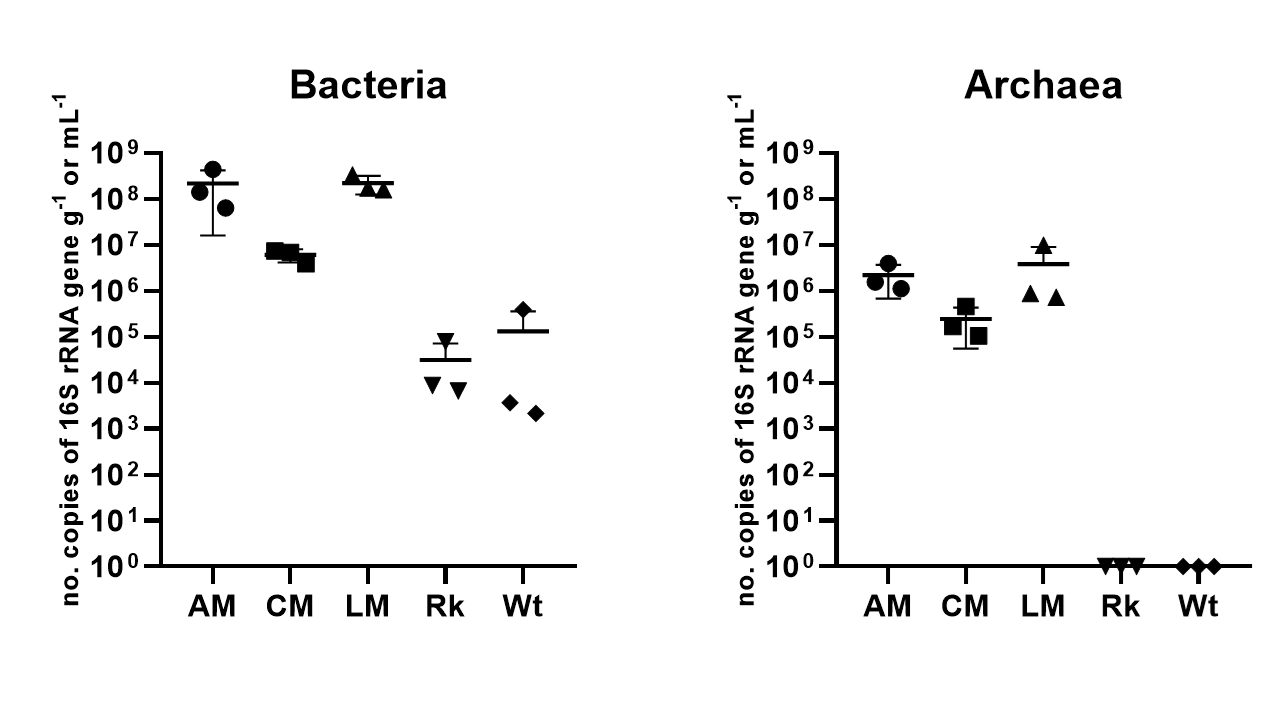


**Fig. S2.** Absolute quantification of the prokaryotic cell biomass present in each cave sample and niche obtained through qPCR experiment targeting specific regions of bacterial and archaeal 16S rRNA gene. The abundance is expressed as number of copies of 16S rRNA per gram or milliliter of sample.

**
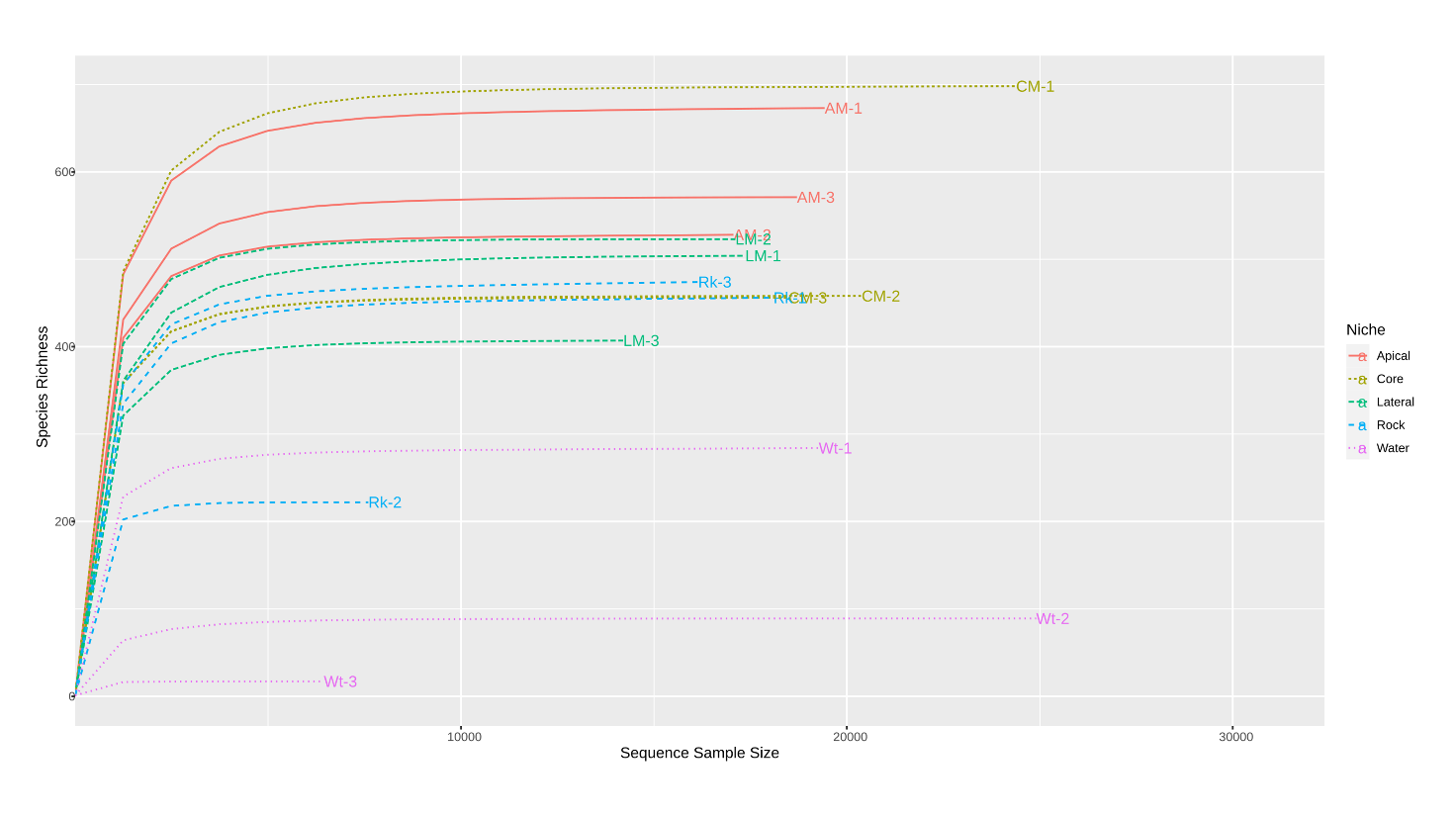
**

**Fig. S3.** Rarefaction curves of the Illumina sequencing data obtained from the fifteen cave samples.


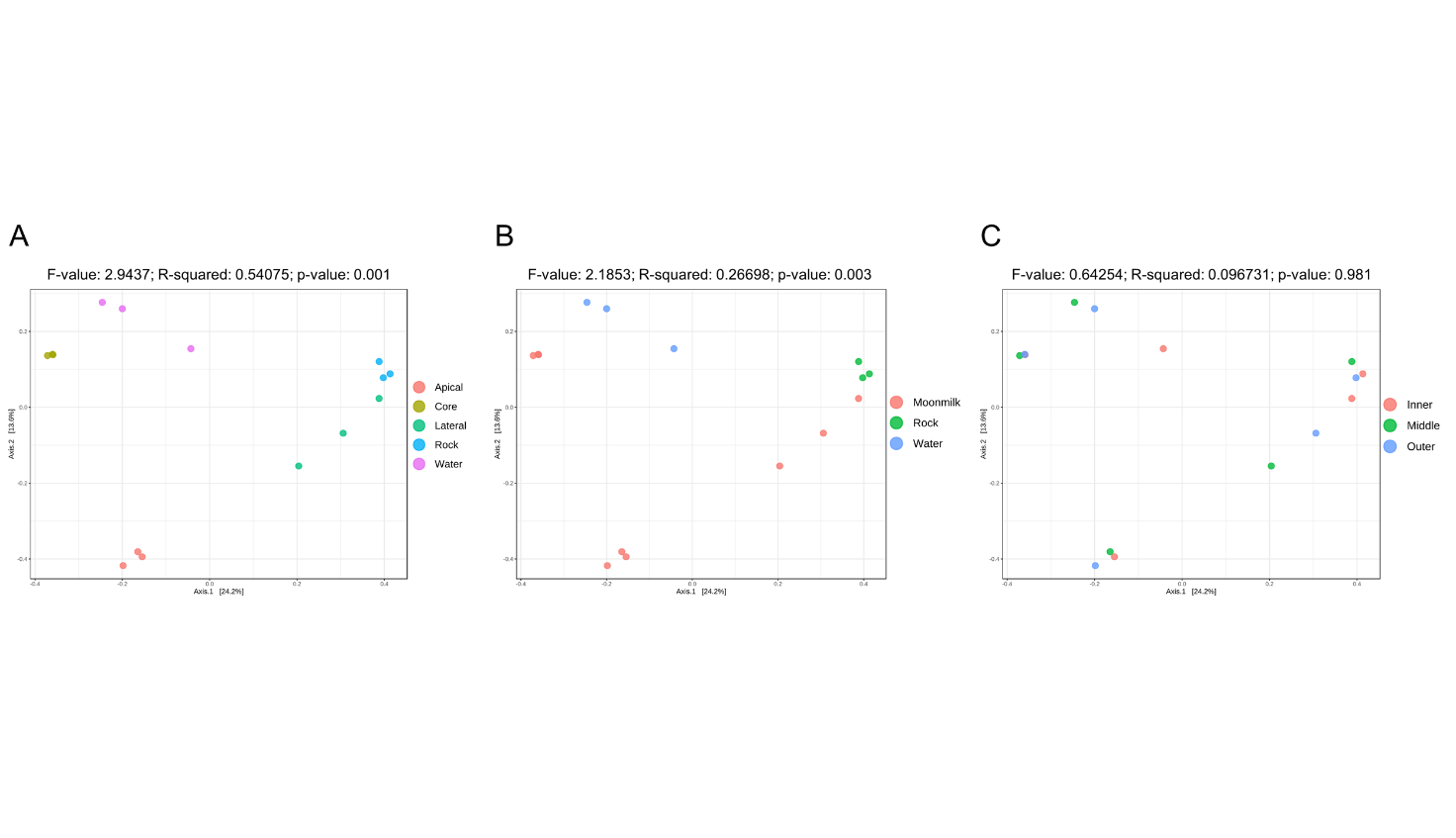


**Fig. S4.** Correlation between the Grotta Nera samples and different environmental parameters. Principal Coordinates Analysis (PCoA) based on Bray-Curtis distance matrix and PERMANOVA statistical method of the microbial communities at ASV level. The environmental parameters considered are the type of matrix/sample dividing the moonmilk into the three parts core, apical and lateral (A) or considering it all together (B), and the cave location (C).


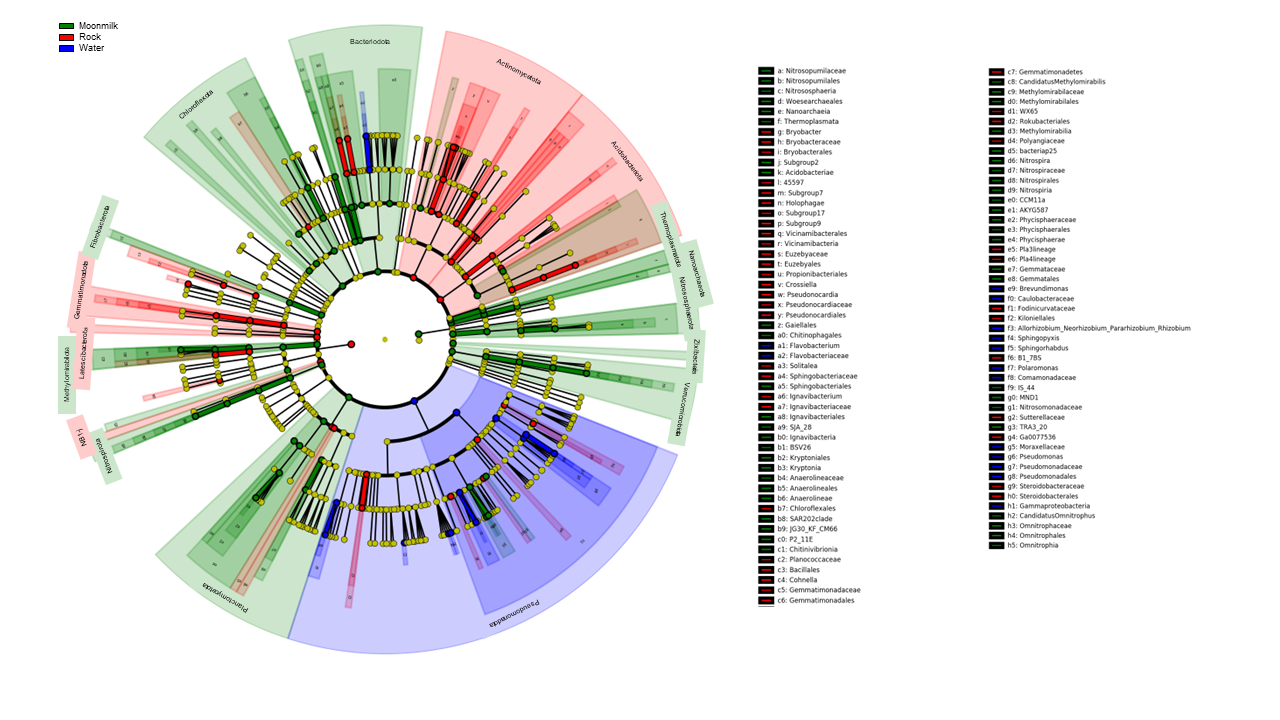


**Fig. S5.** Cladograms showing the differences present between moonmilk, bedrock and waters samples in terms of microbial taxa present according to LEfSe analysis with a LDA threshold of 3.5 and multiclass analysis strategy one-against-all. Taxa with significant differences are highlighted by colored circles and shadings.


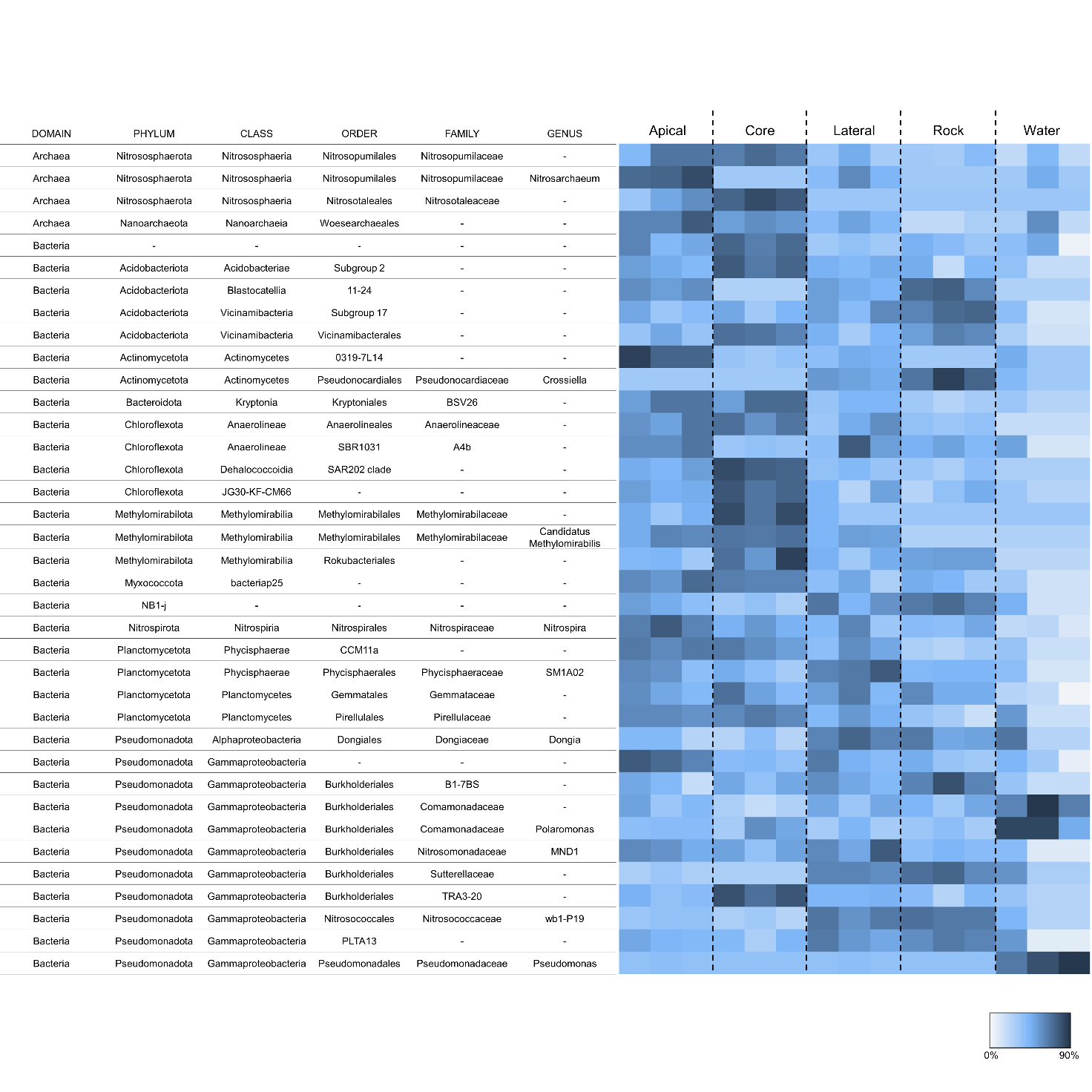


**Fig. S6.** Heatmap showing the abundance of the dominant prokaryotic taxa (with abundance > 1% in each sample group) detected in all the Grotta Nera samples grouped under each corresponding niche.


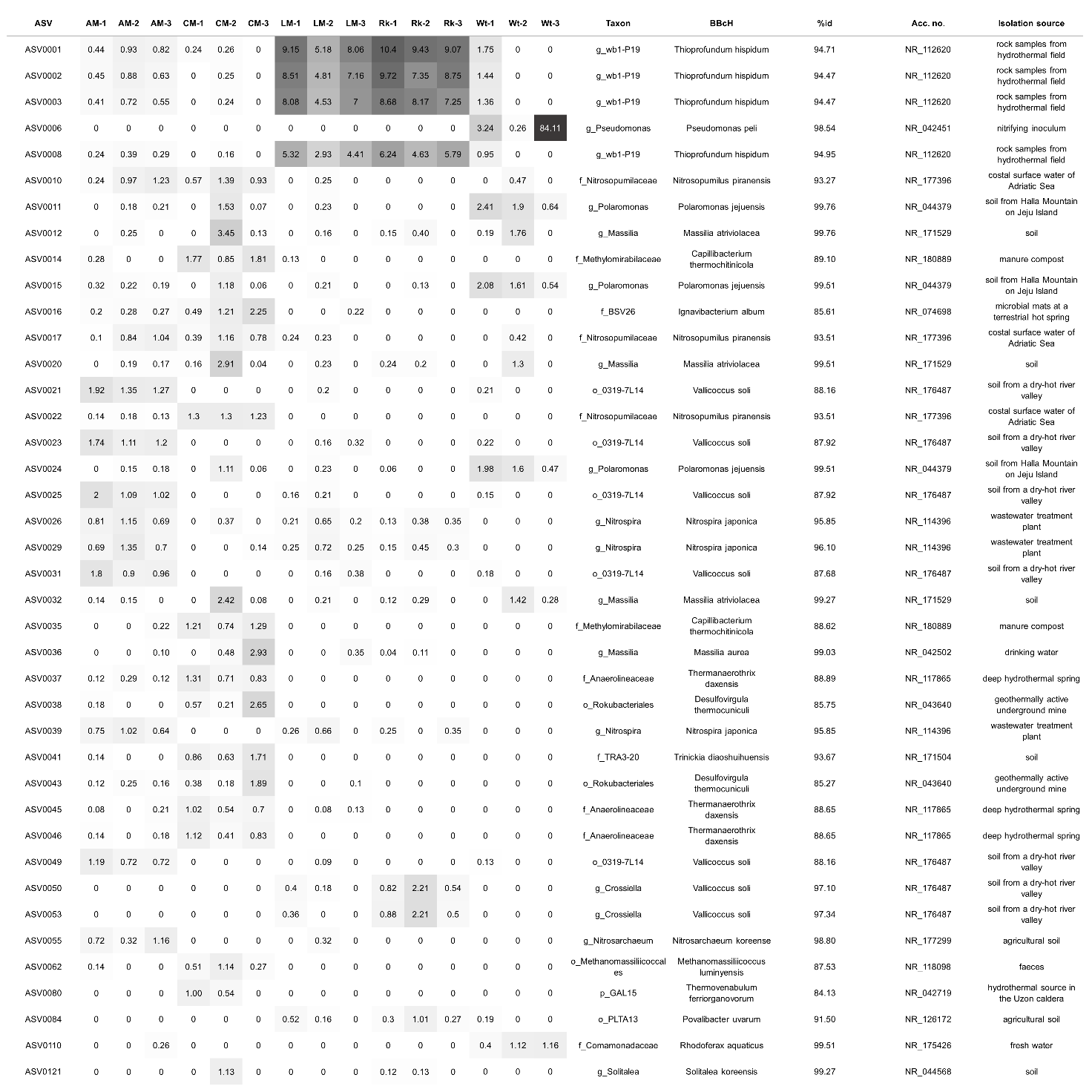


**Fig. S7.** Heatmap showing the abundance and taxonomy affiliation of the dominant ASVs in Grotta Nera samples. The Best Blast classified Hit (BBcH) from NCBI, the identity percentage, the accession number, and the isolation source retrieved from NCBI 16S rRNA sequences database are also reported.


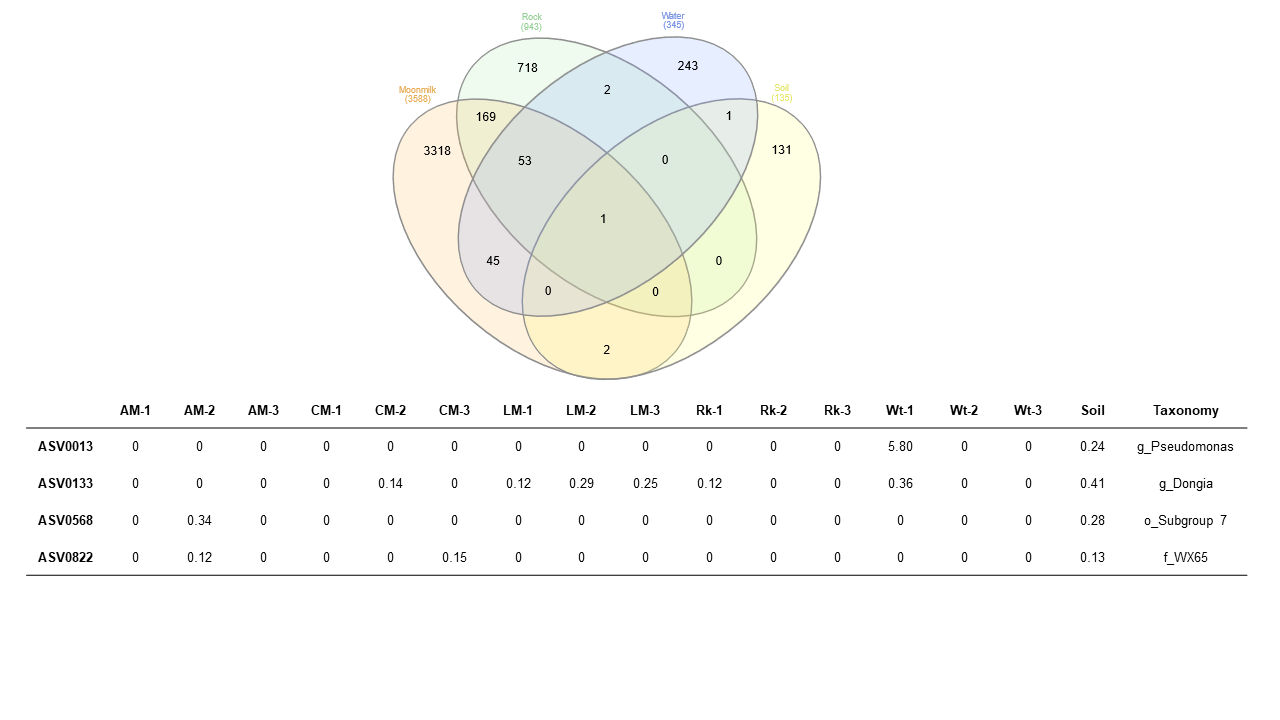


**Fig. S8**. A) Venn diagram illustrating the ASVs that are shared between the moonmilk, rock, water, and soil samples. The ASVs that are shared between the soil and all the other samples are depicted in panel B) together with their abundance (% of the total community) in each sample and the taxonomy.


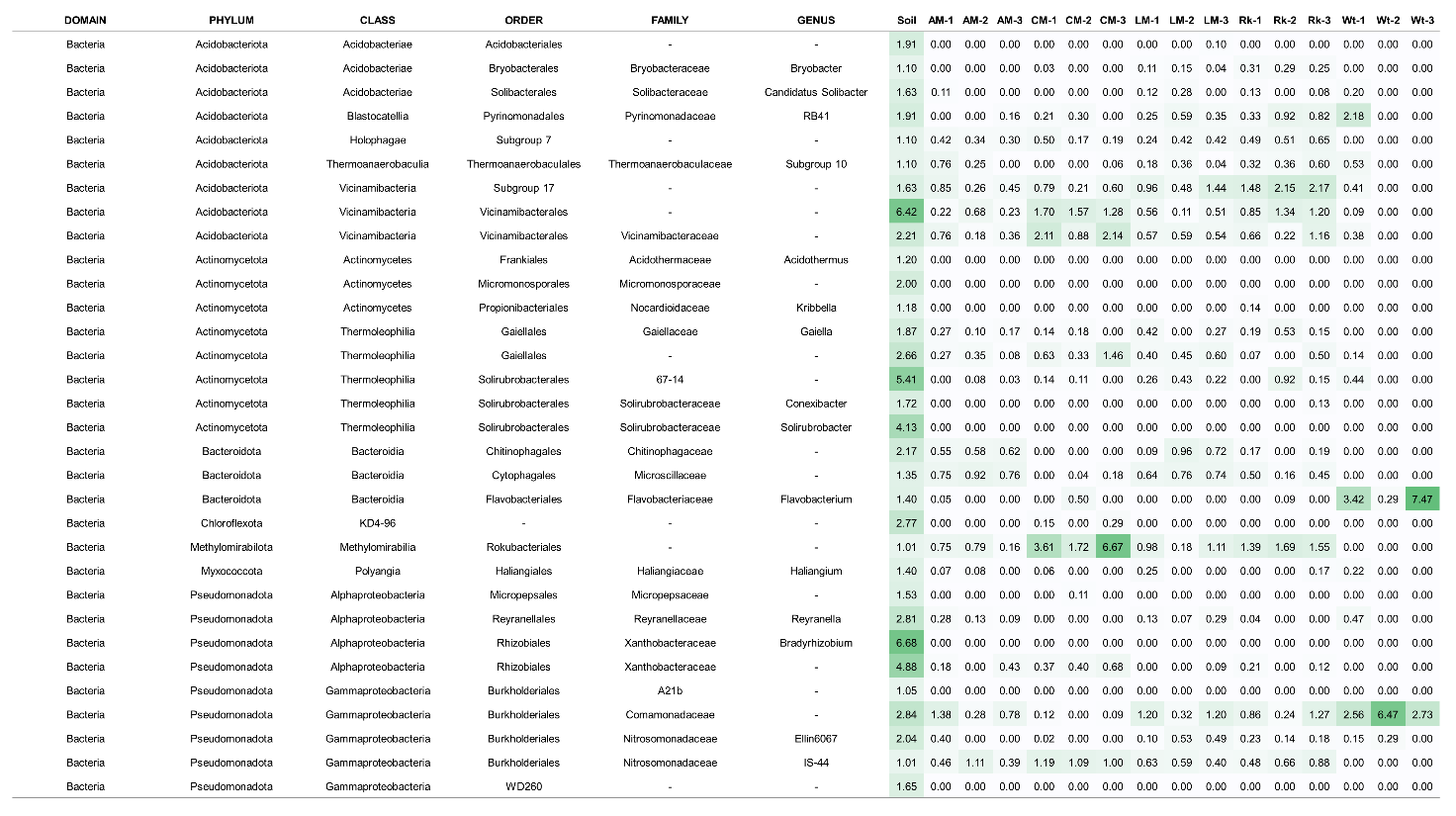


**Fig. S9.** Heatmap showing the distribution and abundance of the soil dominant taxa (> 1% in the soil) over the Grotta Nera samples.
